# Lanthanide Photoluminescence in Heterometallic Polycyanidometallate-Based Coordination Networks

**DOI:** 10.3390/molecules22111902

**Published:** 2017-11-04

**Authors:** Szymon Chorazy, Maciej Wyczesany, Barbara Sieklucka

**Affiliations:** Faculty of Chemistry, Jagiellonian University in Kraków, Gronostajowa 2, 30-387 Kraków, Poland; maciej.wyczesany@student.uj.edu.pl (M.W.); barbara.sieklucka@uj.edu.pl (B.S.)

**Keywords:** functional materials, crystal engineering, coordination networks, poly-cyanidometallates, lanthanides, photoluminescence

## Abstract

Solid-state functional luminescent materials arouse an enormous scientific interest due to their diverse applications in lighting, display devices, photonics, optical communication, low energy scintillation, optical storage, light conversion, or photovoltaics. Among all types of solid luminophors, the emissive coordination polymers, especially those based on luminescent trivalent lanthanide ions, exhibit a particularly large scope of light-emitting functionalities, fruitfully investigated in the aspects of chemical sensing, display devices, and bioimaging. Here, we present the complete overview of one of the promising families of photoluminescent coordination compounds, that are heterometallic d–f cyanido-bridged networks composed of lanthanide(3+) ions connected through cyanide bridges with polycyanidometallates of d-block metal ions. We are showing that the combination of cationic lanthanide complexes of selected inorganic and organic ligands with anionic homoligand [M(CN)_x_]*^n^*^−^ (*x* = 2, 4, 6 and 8) or heteroligand [M(L)(CN)_4_]^2−^ (L = bidentate organic ligand, M = transition metal ions) anions is the efficient route towards the emissive coordination networks revealing important optical properties, including 4f-metal-centred visible and near-infrared emission sensitized through metal-to-metal and/or ligand-to-metal energy transfer processes, and multi-coloured photoluminescence switchable by external stimuli such as excitation wavelength, temperature, or pressure.

## 1. Introduction

Luminescent materials, that are able to emit the light due to absorption of photons, electric current, chemical reactions, or a mechanical action, are applied in the numerous aspects of science, technology, and everyday life. They are utilized in cathode ray or fluorescent tubes, X-ray detectors, lighting and display devices, optical communication, low-energy scintillation, optical storage, light conversion, photovoltaics, chemical sensing, bioimaging, and molecular thermometry [1,2,3,4,5,6]. Considering the construction of light-emitting devices, there are several particularly desired optical functionalities, including white-light emission (WLE), multi-coloured tunable emission, long-lived near-infrared (NIR) phosphorescence, and the non-linear optical property of up-conversion luminescence (UCL) [7,8,9,10]. These functionalities are efficiently realized by the photo- and electroluminescent materials, which are the most attractive in the application aspects [1,2,3,4,5,6].

Among the functional photoluminescent materials, the prime role has been played by traditional inorganic solids, such as oxides, fluorites, and silicates, that can incorporate emissive metal centres, mainly trivalent lanthanide ions revealing a wide range of photoluminescence ranging from UV for Gd^3+^, through visible for Eu^3+^ or Tb^3+^, to NIR light for Nd^3+^ or Yb^3+^ [2]. Photoluminescence was also broadly investigated for organic molecules, especially those built of the expanded system of aromatic rings [1]. Organic chromophores were applied for the preparation of luminescent lanthanide complexes where the organic part serves as an antenna for sensitization of 4f-centred emission through the energy transfer process [11]. Lately, the increasing interest is devoted to the luminescent metal-organic frameworks (MOFs), that belong to the broader group of luminescent coordination polymers (CPs) [12,13,14]. MOFs are composed of metal ions or clusters bonded by organic linkers into the three-dimensional coordination networks, while CPs are generally built of metal centres and organic ligands combined infinitely in at least one direction. Metal-organic frameworks have been intensively investigated in the context of their highly porous character of a potential application in hydrogen storage, selective gas adsorption, and gas sensing [15,16]. More importantly, MOFs can reveal diverse ligand-based, metal-based, charge transfer, or guest-induced photoluminescence due to their multi-component structure [13].

As opposed to classical inorganic solid luminophors, the synthesis of luminescent MOFs can be straightforward, often without applying high temperature or pressure. Their structures are predictable, and can be rationally design through a molecular building block approach. They are transformable into the nanoscale, and can be post-synthetically modified by thermal or chemical treatment. Luminescent MOFs were also found to be efficient platforms for multifunctionality by fruitful addition of magnetic, optical or catalytic functionalities [16]. All these features made MOFs attractive as the luminescent objects, particularly appealing in the aspects of chemical sensing, biomedicine and light-emitting devices [12,13,14]. Of a special interest are lanthanide-based MOFs which were found to exhibit a large scope of luminescent effects, including white-light and tunable visible emission, as well as NIR and up-conversion luminescence [17,18,19,20].

All the positive characteristics of luminescent MOFs can be similarly ascribed to the specific group of coordination systems based on polycyanidometallates. They are constructed of the anionic polycyanidometallate complexes of transition metal ions which are combined through cyanide (CN^−^) molecular bridges with the cationic complexes of various 3d/4d/4f metal ions. Depending on the synthetic conditions, and the addition of supporting organic ligands, they are able to form three-dimensional coordination networks, similar to those characteristic of typical MOFs, but also they can give two- or one-dimensional coordination polymers, and discrete polynuclear clusters of diverse topologies [21,22,23,24]. Cyanide bridges offer the close and strong connection between two different neighbouring metal centres which was fruitfully explored in the induction of strong exchange magnetic coupling [25,26]. This feature is attractive for the photoluminescent properties as the short inorganic CN^−^ linker can strongly couple two metal centres inducing the efficient metal-to-metal energy transfer process. This sounds promising considering the reported polycyanidometallates of 3d/4d/5d metal ions which were found to be strong absorbers of UV light, exhibiting often weak absorption in the visible and NIR ranges. Such spectroscopic behaviour makes them the great candidates for the sensitization of luminescent trivalent lanthanide ions. In addition, some of the polycyanidometallates of transition metal ions reveal the intrinsic photoluminescent properties related to the metal-centred (MC) d-d transitions, the metal-to-ligand charge transfer (MLCT), or the opposite ligand-to-metal charge transfer (LMCT) effects [27,28,29,30,31,32,33].

Heterometallic polycyanidometallate-based coordination networks have been extensively studied in the last decades due to their extraordinary magnetic properties, including strong exchange coupling, long-range magnetic ordering, and strong magnetic anisotropy [25,26,34,35,36,37]. The other fascinating physical functionalities, such as photoinduced magnetic and magneto-optical phenomena, microporosity, catalytic activity, ferroelectricity, and ionic conductivity have been found for the cyanido-bridged coordination compounds [38,39,40,41,42]. Primarily, these properties were investigated within the classical group of three-dimensional bimetallic coordination frameworks based on hexacyanidometallates, called Prussian Blue Analogues (PBAs) [43]. However, the last several years brought the increasing attention pointed to the heterometallic coordination architectures based on other polycyanidometallates, including the homoligand tetracyanido- metallates of Pt^II^/Pd^II^, heptacyanidometallates of Mo^III^/Re^IV^, and octacyanidometallates of Mo^IV/V^, W^IV/V^, Re^V^, Nb^IV^, and the heteroligand [M(L)_x_(CN)_y_]*^n^*^−^ cyanide complexes with blocking organic ligand, L [44,45,46,47,48,49]. As a result, a variety of the [M(CN)_x_]*^n^*^−^-based heterometallic coordination networks were reported, and many extraordinary magnetic, optical, and magneto-optical effects have been presented [50,51,52,53,54]. Some of the polycyanidometallates were also employed in the construction of luminescent functional materials, most successfully realized by combining cyanide metal complexes with lanthanide(3+) ions. Thus, in this review, we present the complete overview of luminescent heterometallic cyanido-bridged networks incorporating 4f metal ions with the emphasis on their structural features and optical properties, including sensitized and/or tunable emission. We focus and discuss only on the lanthanide-containing coordination systems based on polycyanido- metallates for which the photoluminescent properties have been recognized. This review covers the literature sources from 1970s, when this scientific area started, to September 2017.

## 2. Dicyanidometallates, [M^I^(CN)_2_]^−^ (M = Ag, Au)

**Figure 1:**
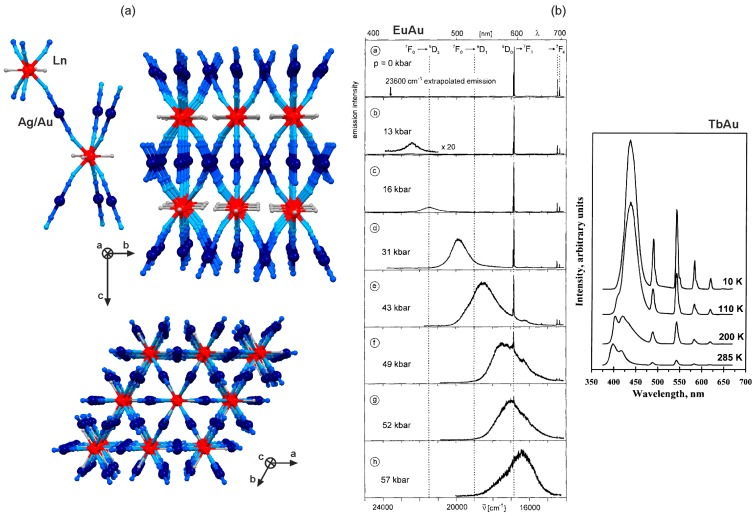
Crystal structure and optical properties of three-dimensional [Ln^III^(H_2_O)_3_][M^I^(CN)_2_]_3_ (Ln = La, Ce, Pr, Nd, Sm, Eu, Gd, Tb, Dy; M = Ag, Au) cyanido-bridged coordination polymers: (**a**) the views of the representative fragment of the structure together with the views of the whole network along *a* and *c* crystallographic directions, (**b**) the low-temperature (*T* = 20 K, *λ*_exc_ = 364 nm) pressure-dependent emission spectra of EuAu network, and the temperature-dependent emission spectra of TbAu compound (*λ*_exc_ = 350 nm). Colours for the structural diagrams: Ln, red; Ag/Au, dark blue; CN^−^, blue; H_2_O, grey [57,58,60]. Reprinted with permission from *Inorg. Chem.* **1998**, *37*, 3209–3316. Copyright 1998 American Chemical Society. Reprinted with permission from *Inorg. Chem.* **2000**, *39*, 4527–4534. Copyright 2000 American Chemical Society.

Thanks to the d^1^° valence electron configuration, dicyanidoargentate(I) and dicyanidoaurate(I) ions are almost colourless as they do not efficiently absorb the visible light. In addition, they are photoluminescent in the visible range due to their metal-to-ligand charge transfer (MLCT) transition [27,28]. They were found to be attractive for the sensitization of lanthanide(3+) luminescence, which was precisely investigated for the family of classical three-dimensional [Ln^III^(H_2_O)_3_][M^I^(CN)_2_]_3_ (Ln = La, Ce, Pr, Nd, Sm, Eu, Gd, Tb, Dy; M = Ag, Au) coordination polymers (Figure 1, Table 1) [55,56,57,58,59,60,61,62,63,64,65,66,67,68,69]. These compounds are composed of the alternately arranged Ln^III^ and M^I^ (M = Ag, Au) centres bonded through cyanide bridges (Figure 1a). Each lanthanide(3+) ion coordinates six cyanides aligned in the nearly trigonal prismatic geometry, and three additional water molecules completing the nine-coordinated metal complex. The whole network is further propagated by the almost linear [M^I^(CN)_2_]^−^ linkages which produce the hexagonal-type of 3D coordination network (Figure 1a) [60]. Under the UV light excitation, dicyanidometallates(I), closely connected with lanthanides, transfer the energy to the 4f metal centre resulting in the sensitized photoluminescence. The significant [M^I^(CN)_2_]^−^ -to-Ln^3+^ energy transfer (ET) process was detected for the Pr-, Eu-, Tb-, and Dy-containing LnAg/LnAu networks [55,56,59,61,65]. For EuAu compound, the efficient ET effect was found under an ambient pressure, and the emission spectrum is dominated by the sharp peaks of Eu^3+^ (Figure 1b). Interestingly, the increase of pressure leads to the shift of the [Au^I^(CN)_2_]^−^-based broad emission towards the lower energies which weakens the spectral overlap with Eu^3+^, and the efficiency of the radiationless energy transfer. Therefore, with increasing pressure, the [Au^I^(CN)_2_]^−^-based luminescent component increases, coexisting with the Eu^3+^ emission, and the complete domination of the broad dicyanidoaurate(I) emission is observed above 50 kbar [57]. The similar effect, but caused by the temperature change, was reported for TbAu analogue. In this material, the cooling procedure leads to the red shift of [Au^I^(CN)_2_]^−^-based emission increasing the spectral overlap with the absorption band of Tb^III^ which produces the enhanced 4f-centred photoluminescence (Figure 1b) [58]. For the lanthanide(3+) ions, which do not reveal the visible emission (La, Nd, Gd), the related 3D LnAg and LnAu networks exhibit the [M^I^(CN)_2_]^−^-centred luminescence, also sensitive to the changes of pressure and temperature [58,62,64]. Moreover, the composition-dependent visible photo- luminescence was presented for the mixed trimetallic LaAgAu and TbAgAu compounds [62,63]. For Ce^3+^ and Sm^3+^, the very weak energy transfer from 4d/5d to 4f metal centres was detected, resulting in the complex UV-Vis emission signal of the mixed Ln^3+^ and [M^I^(CN)_2_]^−^ origins [66,67,68,69]. In the case of Sm^3+^, even the reverse Sm(III) to Au(I) energy transfer could be postulated [69].

**Figure 2:**
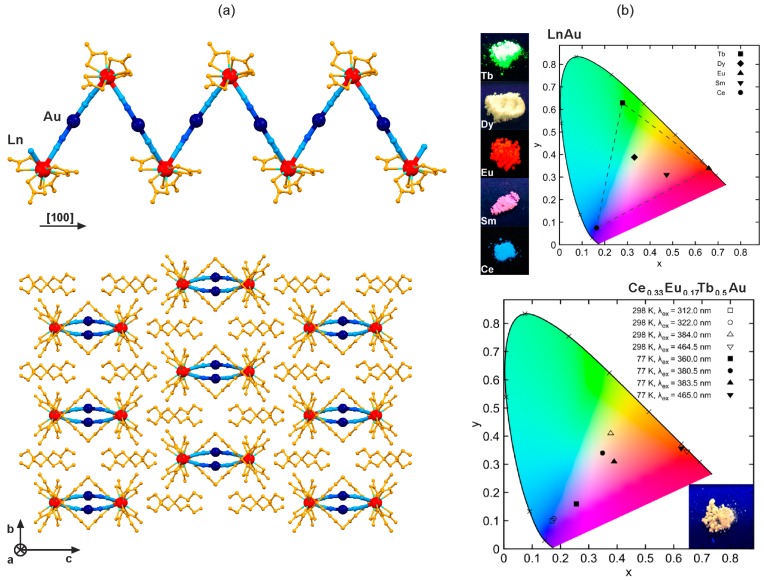
Crystal structure and optical properties of cyanido-bridged (^n^Bu_4_N)_2_[Ln^III^(NO_3_)_4_][Au^I^(CN)_2_] (Ln = Ce, Nd, Sm, Gd, Eu, Tb, Dy) chains: (**a**) the views of the single coordination chain, and the arrangement of chains, (**b**) the multi-coloured lanthanide-dependent emission of LnAu chains together with the related photos under UV light (top), and the multi-coloured temperature- and excitation-dependent emission of Ce_0.33_Eu_0.17_Tb_0.5_Au chains together with the photo under UV light, all presented on the CIE 1931 chromaticity diagram. Colours for the structural diagrams: Ln, red; Au, dark blue; CN^−^, blue; NO_3_^−^ and ^n^Bu_4_N^+^, orange [67,69]. Reprinted with permission from *Inorg. Chem.* **2017**, *56*, 7948–7959. Copyright 2017 American Chemical Society.

The combination of dicyanidoaurates(I) with lanthanide(3+) ions in the non-aqueous solution hampered the crystallization of the 3D network, and gave cyanido-bridged (^n^Bu_4_N)_2_[Ln^III^(NO_3_)_4_] [Au^I^(CN)_2_] (Ln = Ce, Nd, Sm, Gd, Eu, Tb, Dy) zig-zag chains (Figure 2) [67,68,69,70]. For this coordination topology, unlike to the related 3D LnAu networks, there is no efficient energy transfer between [Au^I^(CN)_2_]^−^ and Ln^III^ complexes, and both entities act as two separate emissive sources. This was explained by the spatial separation of the 1D bimetallic LnAu coordination polymers by the [^n^Bu_4_N]^+^ cations. It causes the lack of aurophilic Au–Au interactions (Figure 2a), playing the presumably important role in the ET process within the 3D LnAu networks [67]. Therefore, taking advantage of the intrinsic properties of both luminescent 5d and 4f (Sm, Eu, Tb, Dy) metal complexes embedded in such cyanido-bridged chains, the multi-coloured composition-dependent emission, including the white light, was fruitfully achieved (Figure 2b) [67,68,69,70]. Moreover, the related trimetallic mixed 5d-4f-4f’ compounds enabled the adjustment of the emission colour to the very broad range of the visible spectrum between red colour characteristic of EuAu chains, green emission typical for TbAu chains, up to the deep violet detected for CeAu analogues, for which mainly the UV-violet Ce^III^-centred emission was found (Figure 2b) [68,69]. In addition, the selective excitation of the Au^I^- or Ln^III^-based emission transitions was also achievable, as exemplified by the multi-coloured photoluminescence of the mixed Ce_0.33_Eu_0.17_Tb_0.5_Au chains (Figure 2b) [69].

The further reduction of coordination dimensionality for lanthanide(III)–dicyanidometallates(I) compounds was achieved by the implementation of N,N,N-tridentate 2,2’:6’2”-terpyridine (terpy) ligand. The resulting discrete dinuclear [Ln^III^(terpy)(H_2_O)(NO_3_)_2_][Au^I^(CN)_2_] (Ln = Sm, Eu, Gd, Tb, Dy, Ho, Er, Yb) molecules exhibit the various lanthanide-dependent emission properties [71,72]. In the case of EuAu and TbAu, the dominant visible 4f-centred luminescence was detected due to the efficient intramolecular terpy-to-Ln^3+^ and partial Au^I^-to-Ln^3+^ energy transfer effects. It is accompanied by the residual broad green emission related to the transitions of the [Au^I^(CN)_2_]^−^_2_ dimeric excimers that are formed between the cyanido-bridged molecules. The Au^I^-based emission is dominant for the other lanthanides where the ET processes to 4f metal centre are much less favoured [72]. In GdAu analogue involving Gd^3+^ ion, not luminescent in the visible range, the [Au^I^(CN)_2_]^−^_2_-based green emission is also observed but the emission spectrum is dominated by the other intense red photoluminescence, presumably assigned to the formation of an excited state exciplex of the closely stacked intermolecular terpy ligands [71].

The interesting metal-metal-ligand interactions were observed in the series of coordination systems built of dicyanidoaurate(I) ions, and lanthanide(III) complexes with N,N,N-tridentate bis(benzimidazole)pyridine (bpp) ligands [73]. Depending on the synthetic conditions, the cyanido-bridged tetranuclear [Ln^III^(bbp)_2_][Au^I^(CN)_2_]_3_·2MeCN (Ln = Eu, Gd) molecules, or linear [Ln^III^(bbp)(NO_3_)_2_][Au^I^(CN)_2_]·MeCN (Ln = Eu, Gd, Tb) chains were obtained. For Eu- and Tb-containing species the dominant 4f-centred photoluminescence was achieved, and the efficient bbp-to-Ln^3+^ energy transfer was postulated, while the [Au(CN)_2_]^−^ ions are rather optically silent in this particular case. However, due to the present Au–Au interactions observed between the tetranuclear {LnAu_3_} molecules, the violet [Au(CN)_2_]^−^-based emission could be detected but only for the GdAu analogue. Such 5d-centred photoluminescence was not observed for the cyanido-bridged LnAu chains lacking of the aurophilic interactions. All the related Gd-containing materials show additionally green emission of the bbp ligands. Photoluminescent dicyanidometallate-based coordination systems involving lanthanide ions are summarized in Table 1.

## 3. Tetracyanidometallates, [M^II^(CN)_4_]^2−^ (M = Ni, Pd, Pt)

Among the square planar tetracyanidometallates(II) of group 10 metals, the tetracyanido- platinate(II) ion arouses the greatest interest in the construction of photoluminescent materials as it reveals the strong visible emission. It originates from the metal-to-metal-to-ligand charge transfer (MMLCT) transitions of the one-dimensional [Pt^II^(CN)_4_]^2−^ stacks controlled by the short Pt–Pt contacts [29]. As this emission is closely related to the alignment of square planar metal complexes, it was found to be strongly anisotropic, and the change of the light polarization induces the drastic 3change in the intensity of a few emission components assignable to the various electronic transitions. As a result, the colour of [Pt^II^(CN)_4_]^2−^ photoluminescence is polarization-dependent, and varies in the broad range from approximately blue to red.

**Figure 3:**
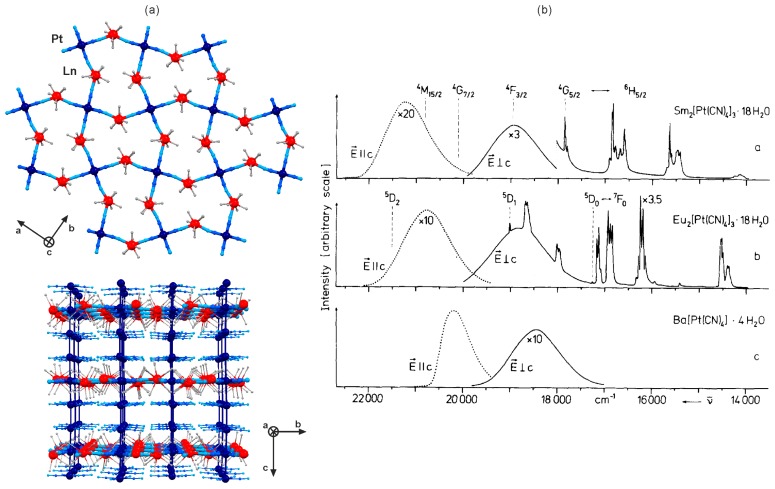
Crystal structure and optical properties of [Ln^III^(H_2_O)_6_]_2_[Pt^II^(CN)_4_]·2{Pt^II^(CN)_4_}·9H_2_O (Ln = La–Lu) layered cyanido-bridged networks: (**a**) the views of the single coordination layer, and the arrangement of the layers and the interlayer [Pt^II^(CN)_4_]^2−^ counterions, (**b**) the polarization- and lanthanide-dependent emission spectra of SmPt, EuPt and the related reference BaPt networks (*T* = 80 K, *λ*_exc_ = 364 nm). Colours for the structural diagrams: Ln, red; Pt, dark blue; CN^−^, blue; H_2_O, grey; Pt-Pt interaction, dark blue stick [74,77]. Adapted from *J. Chem. Phys.* **1978**, *68*, 4707–4713, with the permission of AIP Publishing.

This phenomenon was nicely investigated for the family of classical cyanido-bridged [Ln^III^(H_2_O)_n_]_2_[Pt^II^(CN)_4_]·2{Pt^II^(CN)_4_}·9H_2_O (*n* = 6, Ln = La–Lu; *n* = 5.5, Ln = Eu, Tb) networks (Figure 3, Table 2) [74,75,76,77,78,79]. They are constructed of the coordination layers of a 6-membered metal rings topology based on Pt^II^ and Ln^III^ centres bridged by cyanide ligands within the *ab* plane (Figure 3a). The cyanido-bridged layers are further connected by the close Pt–Pt interactions giving the 1D [Pt^II^(CN)_4_]^2−^ stacks arranged along *c* crystallographic axis. For most of the lanthanide(3+) ions, such LnPt networks reveal mainly the strong and polarization-dependent broad emission of the [Pt^II^(CN)_4_]^2−^ stacks [74,75,76,77]. The emission colour and intensity were found to be dependent on the type of accompanied lanthanide(3+) ions due to the influence of the characteristic excited states of 4f metal ions amending the non-radiative processes within the bimetallic networks [75,77]. Moreover, the LuPt network exhibits the strong impact of the applied magnetic field on the Pt^II^-based photoluminescence due to the role of 4f orbitals of Lu^III^, while the other lanthanides show only very weak emission changes upon the variable magnetic field [76].

In contrast to the other 4f metal ions, SmPt and EuPt networks reveal the strong lanthanide photoluminescence in the visible range indicating the efficient [Pt^II^(CN)_4_]^2−^-to-Ln^3+^ energy transfer mechanism (Figure 3b) [75,77,78,79]. The polarization-dependent Pt^II^-based green to orange emission is still observed but significantly decreased through the radiationless energy transfer. The sensitization process towards Sm^3+^ and Eu^3+^ was reported to be sensitive to the hydrostatic pressure. Its increase induces the red shift of the Pt^II^-centred emission reducing the spectral overlap with Ln^3+^ absorption states that leads to the gradual disappearance of the 4f-centred emission [78,79]. The similar effect is caused by the increase of temperature, clearly hampering the lanthanide photoluminescence [79]. The strong influence of the Pt–Pt distances on the optical properties was proved by comparison of the closely related [Er^III^(H_2_O)_6_]_2_[Pt^II^(CN)_4_]·2{Pt^II^(CN)_4_}·9H_2_O and [Er^III^(H_2_O)_5_(SO_4_)]_2_[Pt^II^(CN)_4_]·{Pt^II^(CN)_4_}·1.5H_2_O networks [74]. They differ in the amount of [Pt^II^(CN)_4_]^2−^ ions occupying the space between the cyanido-bridged layers of an identical 6-membered metal rings topology. The sulfate-containing network shows the smaller number of tetracyanidoplatinates(II) ions resulting in the longer Pt–Pt distances within the one-dimensional metal-metal stacks which induces the significant blue shift of the polarization-dependent Pt^II^-centred emission [74].

**Figure 4:**
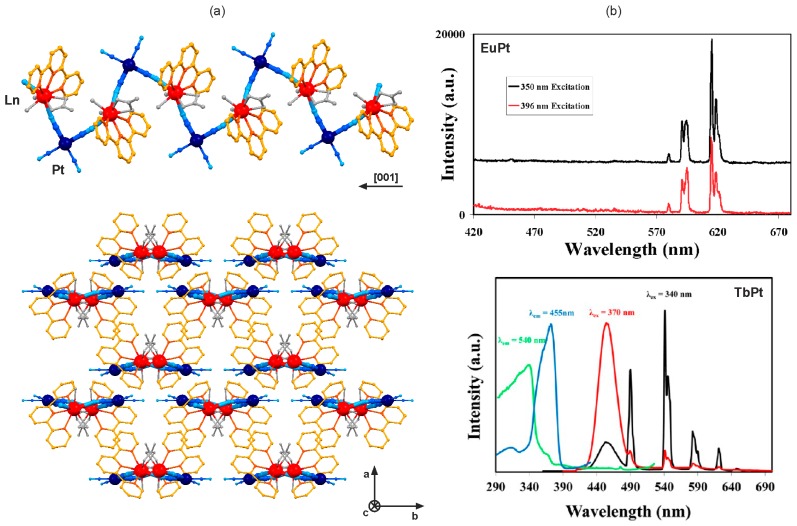
Crystal structure and optical properties of [Ln^III^(terpy)(H_2_O)_2_(NO_3_)][Pt^II^(CN)_4_]·*n*(solvent) (Ln = Eu, Tb; terpy = 2,2’:6’2”-terpyridine) cyanido-bridged chains: (**a**) the views of the single coordination chain, and the arrangement of chains, (**b**) the low temperature (*T* = 77 K) excitation-variable emission spectra for EuPt and TbPt compounds, with the related excitation spectra shown for TbPt. Colours for the structural diagram: Ln, red; Pt, dark blue; CN^−^, blue; terpy, orange; NO_3_^−^ and H_2_O, grey. [81,87] Reprinted with permission from *Inorg. Chem.* **2008**, *47*, 1895–1897. Copyright 2008 American Chemical Society. Reprinted with permission from *Inorg. Chem.* **2012**, *51*, 12230–12241. Copyright 2012 American Chemical Society.

Following the promising optical properties of 3D Ln^III^–[Pt^II^(CN)_4_]^2−^ networks, a considerable number of the related d–f cyanido-bridged coordination polymers revealing photoluminescent functionalities were reported (Table 2) [80,81,82,83,84,85,86,87,88]. The insertion of 2,2’:6’2”-terpyridine (terpy) into the self-assembled Ln^III^–Pt^II^ system resulted in the one-dimensional [Ln^III^(terpy)(H_2_O)_2_(NO_3_)][Pt^II^(CN)_4_]·*n*(solvent) (Ln = Eu, Tb) zig-zag chains (Figure 4a) [81,87]. Under UV light excitation of various wavelengths, the EuPt chains exhibits exclusively the sharp emission peaks assigned to Eu^III^ which clearly indicates the efficient dual acceptor–donor intramolecular energy transfer from both terpy ligand and [Pt^II^(CN)_4_]^2−^ ions to 4f metal centre (Figure 4b) [81]. On the contrary, the TbPt chains shows the dual visible photoluminescence, the green 4f-centred and the blue [Pt^II^(CN)_4_]^2−^- based emission. Thus, only the terpy-to-Tb^3+^ energy transfer is efficient while the analogous Pt^II^-to-Tb^3+^ process is partial. It leaves two distinguishable emission sources that can be selectively induced, giving the excitation-dependent visible photoluminescence (Figure 4b) [87]. The crucial role of direct coordination of both terpy and [Pt^II^(CN)_4_]^2−^ ions to the 4f metal centre was precisely investigated for the Eu^III^–terpy–Pt^II^ system, for which three different crystalline phases, two cyanido-bridged chains, a zig-zag-type [Eu^III^(terpy)(H_2_O)_2_(NO_3_)][Pt^II^(CN)_4_]·MeCN, and a ladder-type [Eu^III^(terpy)(H_2_O)_3_]_2_ [Pt^II^(CN)_4_]_3_·2H_2_O, along with an ionic salt, [Eu^III^(terpy)(H_2_O)_2_(CH_3_COO)_2_]_2_·{Pt^II^(CN)_4_}·3H_2_O, were obtained. For the EuPt one-dimensional coordination polymers, both terpy and cyanide complex are directly bonded to Eu^III^ giving the efficient energy transfer, while the ionic EuPt salt reveals only terpy-to-Eu^3+^ energy transfer, with the additional green luminescence related to the non-coordinated [Pt^II^(CN)_4_]^2−^ ions [82]. The similar effect was also presented for the Tb^III^–Pt^II^ systems with terpy and its 4’-chloro-2,2’:6’2”-terpyridine (terpyCl) derivative. The related cyanido-bridged chains, bearing the directly bonded organic ligand, 4f and 5d metal centres, reveal at least partial dual terpy- and Pt^II^-to-Tb^3+^ energy transfers while the ionic salts with non-coordinated tetracyanidoplatinates(II) show the green Tb^3+^ emission sensitized only by terpy, and the other coordinated ligands [87].

The MMLCT emission of [Pt^II^(CN)_4_]^2−^ ions is strongly determined by the presence of short Pt–Pt contacts. Thus, it is weakened for the compounds lacking of such platinophilic interactions, leaving the possibility to observe the separate 4f- and ligand-centred photoluminescence. Such a situation was found in several tetracyanidometallate-based d–f coordination systems with various organic ligands (Table 2) [80,83,84,85]. For instance, the Ln^3+^ (Ln = Er, Yb) complexes with tetraphenylporphyrinate dianion combined with various tetracyanidometallates resulted in the trinuclear [Ln^III^(tpp)(dmf)_n_]_2_[M^II^(CN)_4_] (Ln = Er, Yb; M = Ni, Pd, Pt) molecules, for which the exclusively red tpp-based emission along with the characteristic Er^3+^ and Yb^3+^ NIR phosphorescence peaks were observed [80]. Similar effects of 4f-centred emission realized by a simple direct f-f excitation or organic ligand-to-Ln^3+^ energy transfer were detected for a series of the ionic salts of lanthanide(3+) ions with dimethylsulfoxide, 1,10-phenanthroline (phen) and 2,2’-bipyridine accompanied by non-coordinated tetracyanidometallates, and for the trinuclear {Ln^III^_2_Pt^II^} molecules with the supporting phen ligand [84,85]. Only in the [Eu^III^(dmf)_2_(terpy)(H_2_O)_2_(NO_3_)]·{Pt^II^(CN)_4_} ionic salt without platinophilic interactions, the weak Pt^II^-based visible emission along with the stronger terpy-sensitized Eu^III^ photoluminescence could be detected [83]. On the other hand, two- dimensional K[Ln^III^(H_2_O)_6_]_2_[Pt^II^(CN)_4_]_3_·{Pt^II^(CN)_4_}·5.5H_2_O (Ln = La, Pr, Nd) honeycomb networks bearing the numerous [Pt^II^(CN)_4_]^2−^ stacks gives the Pt^II^-based green emission, very strong for LaPt, and partially impaired by the excited states of Pr^III^ and Nd^III^ in PrPt and NdPt, respectively [88].

Tetracyanidoplatinate(II) together with dicyanidoaurate(II) were also employed in construction of trimetallic Tb^III^–Pt^II^–Au^I^ coordination networks [86]. The resulting K_2_[Tb^III^(H_2_O)_4_][Pt^II^(CN)_4_]_2_·{Au^I^(CN)_2_}·2H_2_O layers are composed of Tb^III^ bridged by cyanides to Pt^II^ which produces the 2D coordination polymer of an 8-membered metal rings topology. The non-cyanido-bridging [Au^I^(CN)_2_]^−^ moieties are stacked within one type of metal rings, and interact with [Pt^II^(CN)_4_]^2−^ by Pt–Au and Au–Au short contacts. This results in the formation of {Au_2_Pt_4_} metallophilic clusters of their own blue emission. Moreover, these clusters are able to sensitize green Tb^3+^ emission. Upon the addition of 2,2’-bipyridine (2,2’-bpy), the cyanido-bridged heterotrinuclear [Tb^III^(2,2’-bpy)(H_2_O)_4_] [Pt^II^(CN)_4_][Au^I^(CN)_2_]·1.5(2,2’-bpy)·2H_2_O molecules were achieved. The {Tb^III^Pt^II^Au^I^} molecules are also aggregated through Au–Au and Au–Pt interactions, and the tetrameric {Au_2_Pt_2_} metallophilic clusters were detected. This material reveals the green Tb^3+^ emission with {Au_2_Pt_2_}-to-Tb^3+^ energy transfer, and the additional violet 2,2’-bpy emission, proving that [Au^I^(CN)_2_]^−^, [Pt^II^(CN)_4_]^2−^, and their metallophilic clusters can serve as good sensitizers for the lanthanide(3+) photoluminescence [86].

## 4. Hexacyanidometallates, [M^III^(CN)_6_]^3−^ (M = Cr, Co)

**Figure 5:**
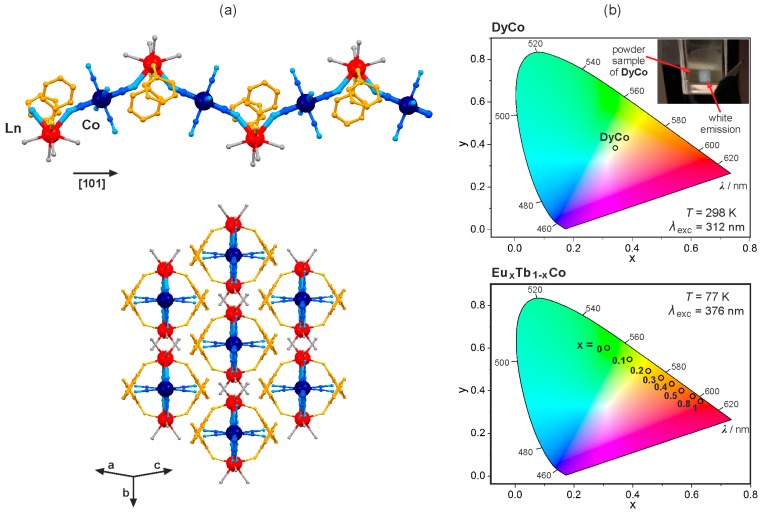
Crystal structure and optical properties of [Ln^III^(3-OHpy)_2_(H_2_O)_4_][Co^III^(CN)_6_]·H_2_O (Ln = Eu, Tb, Dy; 3-OHpy = 3-hydroxypyridine) cyanido-bridged chains: (**a**) the views of the coordination chain, and the arrangement of chains; (**b**) the white light emission of DyCo chains and the related photo (top), the multi-coloured composition-dependent emission of Eu_x_Tb_1-_Co (bottom), all shown on the CIE 1931 chromaticity diagram. Colours for the structural diagram: Ln, red; W, dark blue; CN^−^, blue; 3-OHpy, orange; H_2_O, grey [96,97].

**Figure 6:**
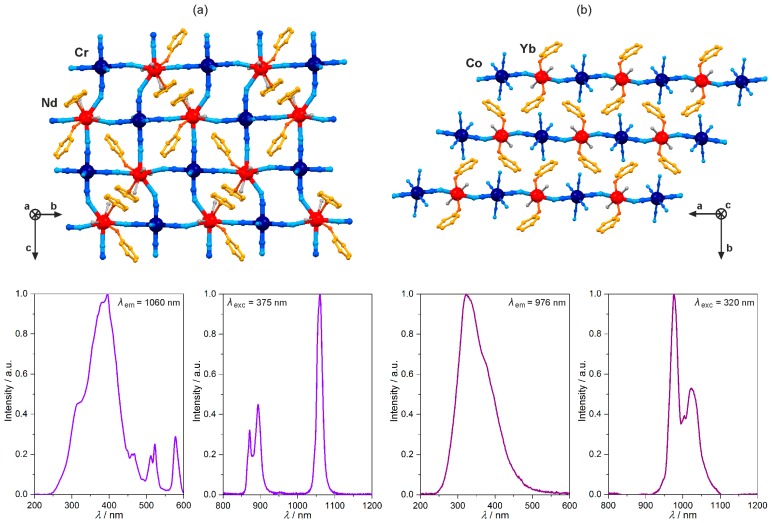
Crystal structure and optical properties of near-infrared (NIR)-emitting cyanido-bridged [Nd^III^(pmmo)_2_ (H_2_O)_3_][Cr^III^(CN)_6_] (**a**) (pmmo = pyrimidine N-oxide) and [Yb^III^(3-pyone)_2_(H_2_O)_2_][Co^III^(CN)_6_] (**b**) (3-pyone = 3-pyridone) coordination polymers. The top part of the figure shows the respective coordination skeletons of square grid layers (**a**) and nearly linear chains (**b**), while the bottom part presents the room temperature excitation and NIR-emission spectra under the indicated wavelength conditions. Colours for the structural diagrams: Nd/Yb, red; Co/Cr, dark blue; CN^−^, blue; pmmo/3-pyone, orange; H_2_O, grey [99,100].

Among the rich diversity of reported hexacyanidometallates [43], only hexacyanido- chromate(III) and hexacyanidocobaltate(III) ions were applied in the construction of photoluminescent d–f coordination networks (Figure 5 and Figure 6, Table 3) [89,90,91,92,93,94,95,96,97,98,99,100]. They reveal the near-infrared and red emission bands, respectively, due to their metal-centred d-d electronic transitions [30,31]. In combination with trivalent lanthanide ions, they form the three-dimensional cyanido-bridged [Ln^III^(H_2_O)_n_][M^III^(CN)_6_]·2H_2_O (M = Cr, Co; *n* = 3, Ln = La–Nd; *n* =2, Nd–Lu) networks with all cyanides bridging to the neighbouring 9- or 8-coordinated 4f metal centres [89,90]. The GdCo and GdCr networks exhibit the d-metal-centred characteristic photoluminescence, observed even at room temperature due to the heavy atom effect [91]. Among the other analogues, the emission properties of the EuCo network was only reported, and red Eu^3+^ photoluminescence sensitized by the Co^3+^-to-Eu^3+^ energy transfer was presented [92,93]. It suggested that [Co^III^(CN)_6_]^3−^ can be a reasonable sensitizer for the visible photoluminescence of 4f metal ions.

Hexacyanidometallates of Co^3+^ and Cr^3+^ exhibit their own emission close to the edge between visible and NIR ranges, so they were expected to be good sensitizers for NIR-emitting lanthanide(3+) ions. It was accurately proved for the cyanido-bridged [Ln^III^(dmf)_4_(H_2_O)_2_][Cr^III^(CN)_6_]·*n*H_2_O (Ln = Nd, Gd, Yb) chains, and dinuclear [Ln^III^(dmf)_4_(H_2_O)_3_][Co^III^(CN)_6_]·*n*H_2_O (Ln = Nd, Gd, Yb) molecules [94,95]. The NdCr, NdCo, YbCr, and YbCo materials show the NIR emission of Nd^3+^ or Yb^3+^, and the lack of the expected d-metal-centred luminescence which indicates the efficient Cr^3+^-to-Ln^3+^ and Co^3+^-to-Ln^3+^ intramolecular energy transfer processes.

The first reports on photoluminescent hexacyanido-bridged d–f materials were expanded by the implementation of various pyrimidine and pyridine derivatives that control the coordination topology [96,97,98,99,100]. The combination of 3-hydroxypyridine (3-OHpy) with Dy^III^ and [Co^III^(CN)_6_]^3−^ produced the cyanido-bridged [Dy^III^(3-OHpy)_2_(H_2_O)_4_][Co^III^(CN)_6_]·H_2_O zig-zag chains (Figure 5a), exhibiting the room temperature white-light Dy^III^ emission realized by the complex excitation involving not only direct f-f transitions, but also Co^3+^-to-Dy^3+^ and 3-OHpy-to-Dy^3+^ energy transfer pathways (Figure 5b) [96]. This topology was also reported for trimetallic [Eu^III^_x_Tb^III^_1-x_ (3-OHpy)_2_(H_2_O)_4_][Co^III^(CN)_6_]·H_2_O materials showing the multi-coloured 4f-centred emission. The colour of luminescence is tuned between red, orange and yellow to green, by the compound’s composition, that is the Eu/Tb ratio governing the intensity of red Eu^3+^ and green Tb^3+^ emission components (Figure 5b) [97]. Multi-coloured emission is also realized for the trimetallic EuTbCo chains by the selection of the appropriate wavelengths of UV light controlling the intensities of Eu^3+^ and Tb^3+^ emission characteristics. Moreover, the 4f-centred photoluminescence is, here, enhanced by the energy transfer occurring from both [Co^III^(CN)_6_]^3−^ and 3-OHpy to lanthanide(3+) ions, and the efficiencies of these radiationless effects play a vital role in the observed switchable emission.

The replacement of 3-hydroxypyridine by 4-hydroxypyridine (4-OHpy) in the Dy^III^–[Co^III^(CN)_6_]^3−^ system resulted in the dramatic change of the structure towards the cyanido-bridged [Dy^III^(4-OHpy)_2_(H_2_O)_3_][Co^III^(CN)_6_]·0.5H_2_O layered framework of a 6-membered metal rings topology. It exhibits the yellow Dy^III^ emission sensitized by the [Co^III^(CN)_6_]^3−^ complex through intramolecular energy transfer. However, in contrast to 3-OHpy, the 4-OHpy ligand is not suitable sensitizer for Dy^III^, and its intrinsic greenish-blue phosphorescence was detected. As a result, the multi-coloured yellow to greenish-blue emission switchable by excitation light, governing the ligand- and Dy^3+^-based luminescent components, was achieved [98]. The layered network but of a square grid topology was prepared by the application of pyrimidine N-oxide (pmmo) with the Nd^3+^ and [Cr^III^(CN)_6_]^3−^ ions (Figure 6a) [99]. The resulting [Nd^III^(pmmo)_2_(H_2_O)_3_][Cr^III^(CN)_6_] material reveals the strong NIR Nd^3+^-centred emission under the UV excitation. The observed excitation spectrum is dominated by the broad complex band assigned to the absorption of pmmo and [Cr^III^(CN)_6_]^3−^ (Figure 6a). This, together with the disappearance of the expected green phosphorescence of organic ligand, and NIR emission of a cyanide complex, proved the efficient energy transfer from both organic and inorganic building blocks. The similar dual donor intramolecular energy transfer related to the direct coordination of the organic ligand and hexacyanidometallate to 4f metal was presented for almost linear [Yb^III^(3-pyone)_2_(H_2_O)_2_][Co^III^(CN)_6_] (3-pyone = 3-pyridone) chains (Figure 6b) [100]. Under the UV excitation, they show typical NIR Yb^3+^ emission, enhanced by the radiationless energy transfer from 3-pyone and [Co^III^(CN)_6_]^3−^ to 4f metal ion, which proves that hexacyanidocobaltate(III) is particularly attractive photoluminescent metalloligand being a good sensitizer for both visible light- and NIR-emitting lanthanide(3+) ions.

## 5. Octacyanidometallates, [M^IV/V^(CN)_8_]^4−/3−^ (M = Mo, W)

**Figure 7:**
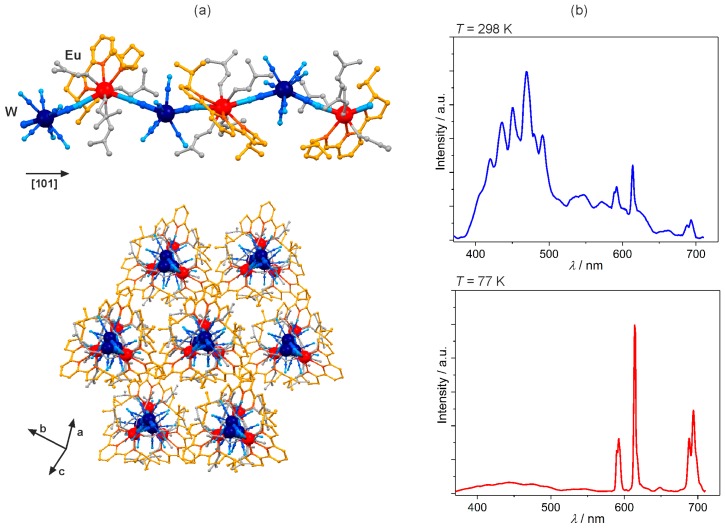
Crystal structure and optical properties of chiral [Eu^III^(*RR*-*i*Pr-pybox)(dmf)_4_][W^V^(CN)_8_]·dmf·8H_2_O (*RR*-*i*Pr-pybox = *RR*-2,2’-(2,6-pyridinediyl)bis(4-isopropyl-2-oxazoline)) cyanido-bridged chains: (**a**) the views of the single chiral helical chain, and the arrangement of chains, (**b**) the temperature-dependent emission spectra collected under the UV light excitation of 340 nm. Colours for the structural diagram: Eu, red; W, dark blue; CN^−^, blue; *RR*-*i*Pr-pybox, orange; dmf, grey [107].

**Figure 8:**
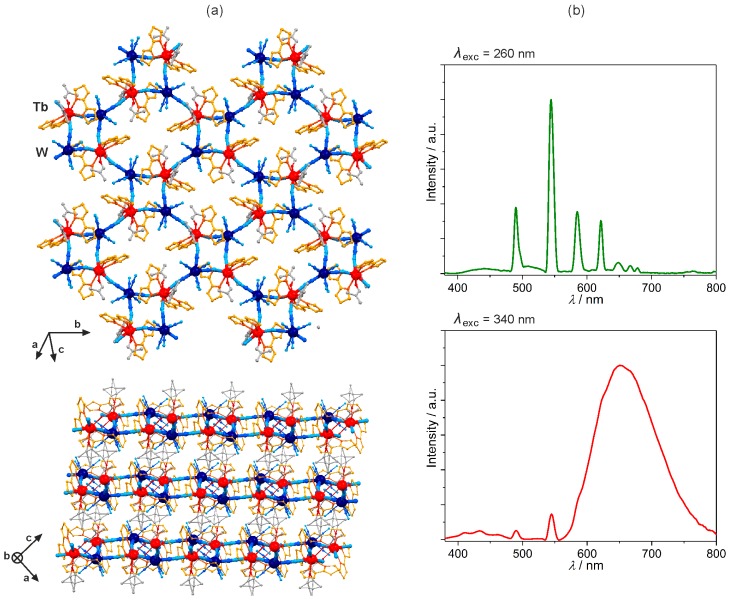
Crystal structure and optical properties of two-dimensional [Tb^III^(box)_2_(dmf)_2_] [W^V^(CN)_8_]·H_2_O (box = 2,2’-bis(2-oxazoline)) coordination polymers: (**a**) the views of the single cyanido-bridged layer, and the arrangement of layers, (**b**) the excitation-dependent emission spectra collected at *T* = 77 K. Colours for the structural diagram: Tb, red; W, dark blue; CN^−^, blue; box, orange; dmf, grey [109].

Octacyanidometallate complexes of Mo(IV,V) and W(IV,V) are not emissive as their numerous d-d and charge transfer (ligand-to-metal or metal-ligand) electronic transitions were found to be rather photoreactive which was utilized in the construction of photomagnetic materials [23]. These cyanide complexes offer, however, very light yellow colour as their absorption bands are shifted to the UV range. It enables the observation of visible and NIR photoluminescence of accompanying emissive chromophores [32]. In effect, a considerable number of photoluminescent d–f coordination networks based on octacyanidometallates were reported (Figure 7 and Figure 8, Table 4) [101,102,103,104,105,106,107,108,109,110]. In the aqueous solution, trivalent lanthanide(3+) and [M^V^(CN)_8_]^3−^ ions produce the cyanido-bridged [Ln^III^(H_2_O)_5_][M^V^(CN)_8_] (Ln = Sm, Eu, Gd, Tb; M = Mo, W) layered frameworks of a square grid topology [101,102,103]. Such EuMo and EuW layers exhibit the specific Eu^3+^ emission realized partially by direct f-f excitation, and the more sophisticated energy transfer from O-Eu (EuMo) or O-W (EuW) ligand-to-metal charge transfer (LMCT) bands situated in the UV range [101]. In the analogous TbMo and TbW compounds, the green emission of Tb^3+^ was detected after the excitation by the UV light, related to the Tb-based d–f and f–f electronic transitions [101,102]. Similar Eu^3+^ or Tb^3+^ emission was reported for the mixed [Eu^III^_0.5_Gd^III^_0.5_(H_2_O)_5_][W^V^(CN)_8_] and [Sm^III^_0.5_Tb^III^_0.5_(H_2_O)_5_][W^V^(CN)_8_] networks. The trimetallic [Eu^III^_0.5_Tb^III^_0.5_(H_2_O)_5_] [W^V^(CN)_8_] framework exhibits both red Eu^3+^ and green Tb^3+^ emission peaks realized by the LMCT, O-Eu or W-related, excitation, and the additional Tb^3+^-to-Eu^3+^ intermetallic energy transfer occurring through the [W^V^(CN)_8_]^3−^ moieties [103]. The emission of Nd^3+^ was also possible to detect within the family of octacyanidometallate-based materials, as reported for the cyanido-bridged [Nd^III^(phen)_2_(dmf)_2_(H_2_O)][Mo^V^(CN)_8_]·2H_2_O and [Nd^III^(phen)(dmf)_5_][M^V^(CN)_8_]·*x*H_2_O (M = Mo, W; phen = 1,10-phenanthroline) chains showing NIR emission of 4f metal ion sensitized by phen ligand [104].

The interesting luminescent functionalites were presented for the three-dimensional hybrid inorganic-organic [Ln^III^_2_(mpca)_2_(MeOH)_2_(H_2_O)_6_][Mo^IV^(CN)_8_]·*x*MeOH (Ln = Nd, Eu, Tb; mpca = 5-methyl-2-pyrazine carboxylic acid) networks where cyanido-bridged layers are connected in the third direction by organic mpca linkers [105]. They reveal the strong 4f-metal centred photoluminescence sensitized by mpca ligands while [Mo^IV^(CN)_8_]^4−^ serves as an inorganic linker stabilizing the 3D coordination network. Moreover, the Eu^3+^ emission was found to be strongly sensitive to the amount of water molecules occupying the interstitial space within the crystal structure. Therefore, the emission intensity of the EuMo network increases significantly with decreasing humidity of the air around the solid sample, which makes this material an efficient humidity sensor working in the whole relative humidity range from 0 to 100% [106].

The implementation of the enantiopure derivatives of 2,2’-(2,6-pyridinediyl)bis(2-oxazoline) (pybox) molecule into the Ln^III^–[W^V^(CN)_8_]^3−^ systems resulted in the formation of chiral cyanido-bridged [Ln^III^(*i*Pr-pybox)(dmf)_4_][W^V^(CN)_8_]·dmf·4H_2_O (Ln = Nd, Eu, Gd; *i*Pr-pybox = (*SS*)- or (*RR*)-2,2’-(2,6-pyridinediyl)bis(4-isopropyl-2-oxazoline); Figure 7a) and [Ln^III^(ind-pybox)(dmf)_4_] [W^V^(CN)_8_]·5MeCN·4MeOH (Ln = Nd, Gd; ind-pybox = (*SRSR*)- or (*RSRS*)-2,6-bis[8H-indeno[1,2-d]oxazolin-2-yl]pyridine) helices [107,108]. Taking advantage of both luminescent Ln^3+^ and pybox ligand, the Eu(*i*Pr-pybox)W chains reveal the thermal switching between red Eu^3+^ emission predominant at low temperature due to the effective ligand-to-metal energy transfer, and blue *i*Pr-pybox-centred photoluminescence prevailing at room temperature, as the energy back transfer to ligand is operating at higher temperatures (Figure 7b) [107]. The GdW helices with *i*Pr-pybox, and the more expanded ind-pybox ligands, exhibit exclusively the ligand-based red phosphorescence. The NdW chains show the 4f-metal-centred NIR emission enhanced by the pybox-to-Nd^3+^ energy transfer process [108].

The rich scope of diverse lanthanides(3+) photoluminescence, and their interaction with organic 2,2’-bis(2-oxazoline) (box) organic chromophore was beautifully presented in the cyanido-bridged [Ln^III^(box)_n_(dmf)_m_][M^V^(CN)_8_]·*x*(solvent) (Ln = Ce–Dy, n = 2, m = 2; Ln = Ho–Yb, n = 1, m = 3; M = Mo, W) layers of a mixed 4- and 8-membered metal rings topology, showing the unusual sliding of coordination layers depending on lanthanides [109,110]. The TbW derivative, named [Tb^III^(box)_2_(dmf)_2_][W^V^(CN)_8_]·H_2_O (Figure 8a), reveals the excitation-dependent visible luminescence switchable between Tb^3+^ green emission induced under the deep UV excitation of the interconfigurational d-f transition of the 4f-metal centre, and red box-based phosphorescence detected for the UV excitation around 340 nm that directs the energy mainly towards the ligand excited states (Figure 8b) [109]. Similar effect was found for the analogous TbMo system while the other members of the LnMo family showed lanthanide-dependent visible and/or NIR emission. For PrMo, SmMo, EuMo and HoMo, the box-to-Ln^3+^ energy transfer induced the 4f-centred visible emission ranging from green for Ho^3+^, orange for Sm^3+^, to red for Pr^3+^ and Eu^3+^. The ligand-to-metal energy transfer is also responsible for the observation of characteristic emission peaks in the NIR range for several compounds, PrMo, NdMo, SmMo, HoMo and YbMo layers [110].

## 6. Heteroligand Tetracyanidometallates, [M^II^(L)(CN)_4_]^2−^ (M = Ru, Os)

**Figure 9:**
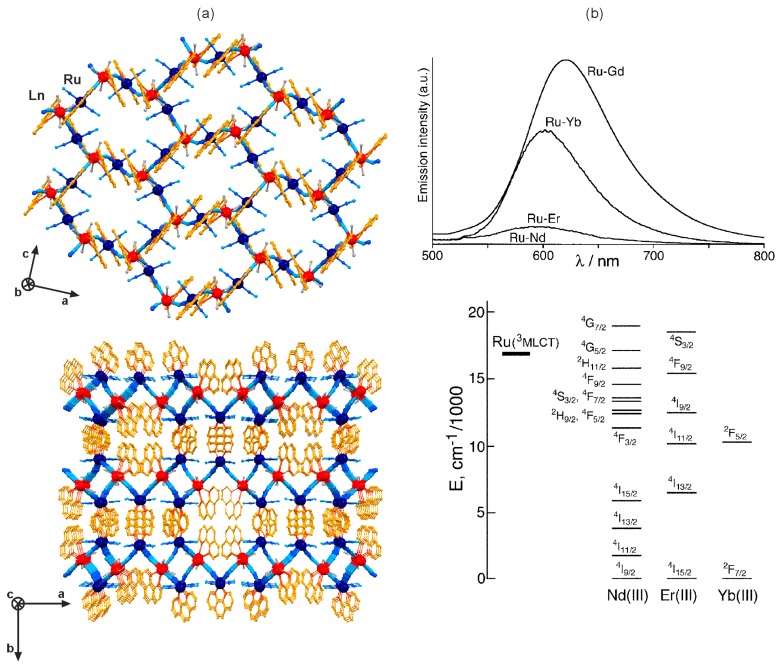
Crystal structure and optical properties of two-dimensional [Ln^III^(phen)(H_2_O)_3_]_2_ [Ru^II^(phen)(CN)_4_]·14H_2_O (Ln = Nd, Gd, Er, Yb; phen = 1,10-phenanthroline) coordination polymers: (**a**) the views of the cyanido-bridged layer, and the arrangement of layers, (**b**) the lanthanide- dependent room temperature emission spectra in the visible range together with the related energy level diagram. Colours for the structural diagram: Ln, red; Ru, dark blue; CN^−^, blue; phen, orange; H_2_O, grey. Adapted from Ref. [114] with permission from The Royal Society of Chemistry.

**Figure 10:**
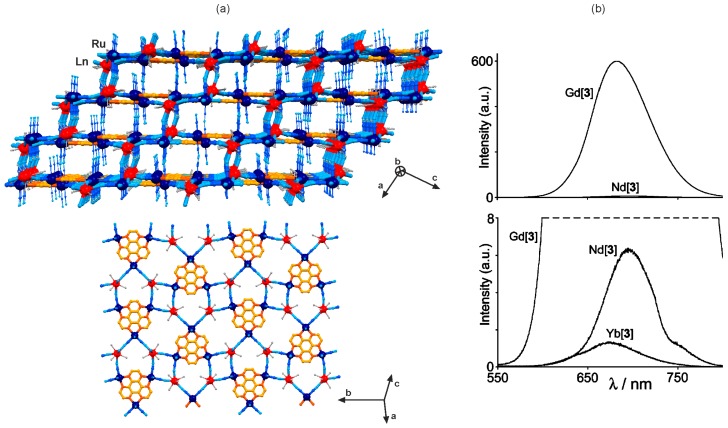
Crystal structure and optical properties of three-dimensional [Ln^III^(H_2_O)_4_]_2_ [{Ru^II^(CN)_4_}_3_(HAT)]·13H_2_O^4^ (Ln = Nd, Gd, Yb; HAT = hexaazatriphenylene) coordination networks: (**a**) the view of the whole network along the *b* crystallographic direction, and the detailed view of the layered fragment of the 3D network situated within the (101) plane, (**b**) the lanthanide-dependent room temperature emission spectra in the visible range. Colours for the structural diagram: Ln, red; Ru, dark blue; CN^−^, blue; HAT, orange; H_2_O, grey [116]. Reprinted with permission from *J. Am. Chem. Soc.* **2007**, *129*, 11491–11504. Copyright 2007 American Chemical Society.

Beside the homoligand polycyanidometallates, [M(CN)_x_]*^n−^* (x = 2, 4, 6, and 8), the photo-luminescent d–f coordination networks were prepared using the heteroligand tetracyanidometallate complexes, [M^II^(L)(CN)_4_]^2−^ (M = Ru, Os), where two positions of the octahedron are blocked by the aromatic N,N-bidentate organic ligands, L [111,112,113,114,115,116,117,118,119]. Depending on the selected L ligand, exemplified by 2,2’-bipyridine (2,2’-bpy), 1,10-phenanthroline (phen), or 2,2’-bipyrimidine (bpym), these heteroligand cyanide complexes of Ru^II^ and Os^II^ reveal the intrinsic green to red emission of the metal-to-ligand charge transfer (MLCT) origin [33]. Due to their energy position close to the red region of the visible spectrum, the MLCT excited states of [M^II^(L)(CN)_4_]^2−^ (M = Ru, Os) ions were found to be suitable for the sensitization of NIR-emitting lanthanide(3+) ions (Figure 9 and Figure 10, Table 5) [111,112,113,114,115,116,117]. It was firstly presented for the yellow emissive [Ru^II^(2,2’-bpy)(CN)_4_]^2−^ anions which combined with 4f metal ions in the aqueous solution produced the cyanido-bridged trinuclear K[Ln^III^(H_2_O)_n_][Ru^II^(2,2’-bpy)(CN)_4_]_2_·9H_2_O (*n* = 7, Ln = Pr; *n* = 6, Ln = Er, Yb) molecules, or the two-dimensional [Ln^III^(H_2_O)_4_]_2_[Ru^II^(2,2’-bpy)(CN)_4_]_3_·*n*H_2_O (Ln = Gd, Nd) layered frameworks of a mixed 4- and 12-membered metal rings topology. While the yellow MLCT emission was detected in GdRu, the NIR 4f-centred emission, enhanced by efficient Ru^2+^-to-Ln^3+^ energy transfer, was observed for PrRu, ErRu, YbRu and NdRu materials. Using the experimentally determined degree of quenching of the Ru^II^-based emission, the rates of metal-to-metal energy transfer could be estimated. The fastest energy transfer was found for NdRu, the slowest for YbRu, while the intermediate values were assigned to PrRu and ErRu, which was rationalized on the basis of the spectral overlap between the available excited f–f states with the Ru^2+^ emission band [112].

The tetracyanidoruthenate(II) complex bearing 2,2’-bipyrimidine (bpym) ligand, [Ru^II^(bpym) (CN)_4_]^2−^, forms with lanthanide(3+) ions two types of coordination polymers. The lighter 4f metal ions of Nd^3+^, Sm^3+^ and Gd^3+^ gave the helical [Ln^III^(H_2_O)_5_(NO_3_)][Ru^II^(bpym)(CN)_4_] chains, while the heavier Er^3+^ and Yb^3+^ produced the cyanido-bridged [Ln^III^_2_(H_2_O)_7.5_(NO_3_)_1.5_][Ru^II^(bpym)(CN)_4_]_2_·5.5H_2_O layers of a complex cross-linked square-based chains topology [113]. The red Ru^II^-based emission was found for GdRu, while all the NIR-emitting Ln^3+^ ions (Nd, Er, Yb) reveal their own photoluminescence, efficiently sensitized by the [Ru^II^(bpym)(CN)_4_]^2−^ moieties, with the better spectroscopic overlap and resulting energy transfer efficiency for ErRu and RuYb when compared with the yellow emissive [Ru^II^(2,2’-bpy)(CN)_4_]^2−^ anions. In addition, a different, expanded dinuclear bpym-bridged [{Ru^II^(CN)_4_}_2_(bpym)]^4−^ anion were prepared, and successfully applied for the construction of heterometallic d–f coordination networks. The hybrid inorganic-organic [Ln^III^(H_2_O)_5_(NO_3_)]_2_[{Ru^II^(CN)_4_}_2_(bpym)]·3H_2_O (Ln = Nd, Sm) ladder chains, and three-dimensional [Ln^III^_1.5_(H_2_O)_7_][{Ru^II^(CN)_4_}_2_(bpym)]·(NO_3_)·*n*H_2_O (Ln = Eu, Gd, Yb) networks of a mixed 2D cyanido-bridged, and an 1D bpym-bridged connectivity, were isolated. They revealed the analogous sensitized near-infrared 4f-metal-centred emission [113].

The bimetallic 4d–4f coordination systems involving [Ru^II^(L)(CN)_4_]^2−^ ions offer a rich structural diversity as the blocking L ligand and four potentially bridging cyanides can result in the various low dimensional molecular systems. Moreover, the large lanthanide(3+) ions reveal high coordination numbers that the supporting organic ligands could be also introduced. It was fruitfully presented for a series of zero-, one- and two-dimensional cyanido-bridged networks based on [Ru^II^(phen)(CN)_4_]^2−^ (phen = 1,10-phenanthroline) anion [114]. Using the phen molecule as the ligand coordinated both to the 4d and 4f metal centres, the cyanido-bridged [Ln^III^(phen)(H_2_O)_3_]_2_ [Ru^II^(phen)(CN)_4_]·14H_2_O (Ln = Nd, Gd, Er, Yb) layers of a corrugated honeycomb topology were obtained (Figure 9a). The GdRu layers reveal the strong MLCT red emission of [Ru^II^(phen)(CN)_4_]^2−^ which is significantly quenched for the analogous NdRu, ErRu, and YbRu coordination frameworks due to the Ru^2+^-to-Ln^3+^ energy transfer process (Figure 9b). The MLCT band completely disappears for NdRu as the high density of low-lying f–f excited states of Nd^3+^ accepting the energy from Ru^II^. For ErRu and YbRu derivatives, the rate of d–f energy transfer, and the resulting the degree of quenching of the MLCT luminescence, decreased due to the smaller number of accessible excited f–f states (Figure 9b) [114]. The similar sensitized NIR emission was found for the other phen- containing cyanido-bridged hexanuclear K_2_[Ln^III^(phen)_2_(H_2_O)]_2_[Ru^II^(phen)(CN)_4_]_4_·*n*(solvent) (Ln = Pr, Nd, Er, Yb) molecules, prepared under the modified synthetic conditions. The application of other ancillary oligopyridine ligands, including 2,2’:6’2”-terpyridine (terpy) and 2,2’-bipyrimidine (bpym), resulted in the formation of one-dimensional cyanido-bridged [Ln^III^(terpy)(H_2_O)_3_]_2_ [Ru^II^(phen)(CN)_4_]_3_·*n*H_2_O (Ln = Pr, Nd, Er, Yb) and [Ln^III^_2_(bpym)(H_2_O)_7_][Ru^II^(phen)(CN)_4_]_3_·*n*solvent (Ln = Nd, Er, Yb) networks of a ladder chain, and a hybrid chain of squares topologies, respectively. They all exhibit the characteristic 4f-metal-centetered NIR emission enhanced by the Ru^2+^-to-Ln^3+^ energy transfer. It was proved that blocking polypyridine ligands hamper the coordination of cyanides and water molecules to the 4f metal ions, not only amending the structural topology but also visibly increasing lanthanide(III)-based emission lifetimes when compared to the networks without these supporting ligands [114].

The particularly efficient sensitization of both Nd^3+^ and Yb^3+^ was achieved for the heterometallic d–f coordination networks based on tetracyanidoruthenates(II) bearing the expanded aromatic system of hexaazatriphenylene (HAT) (Figure 10) [115,116]. The simplest metal-cyanide complex of this family, [Ru^II^(CN)_4_(HAT)]^2−^ was combined with lanthanide(3+) ions giving the hybrid layered [Ln^III^(H_2_O)_5_]_2_[Ru^II^(HAT)(CN)_4_]·*n*H_2_O framework of a 12-membered metal rings topology for larger lanthanides of Nd, Sm, Eu, Gd, or the one-dimensional cyanido-bridged [Yb^III^_2_(H_2_O)_9_(NO_3_)_2_][Ru^II^ (HAT)(CN)_4_]·2(NO_3_)·6.5H_2_O ladders bearing smaller Yb^3+^ ions. Using the other cyanide precursor of [{Ru^II^(CN)_4_}_2_(HAT)]^4−^ with two tetracyanidoruthenates(II) attached to a single HAT, the very different hybrid [Yb^III^_3_(H_2_O)_2_(NO_3_)][{Ru^II^(CN)_4_}_2_(HAT)]_2_·20H_2_O layers of 6-membered metal rings topology were synthesized. The most expanded [{Ru^II^(CN)_4_}_3_(HAT)]^6−^ anion, offering three Ru^II^ centres linked by a single HAT, and 12 potentially bridging cyanide ligands, was mixed with lanthanide(3+) ions producing the three-dimensional [Ln^III^(H_2_O)_4_]_2_[{Ru^II^(CN)_4_}_3_(HAT)]·13H_2_O (Ln = Nd, Gd, Yb) pillared network. They are constructed of the hybrid HAT-cyanide heterometallic layers connected in the perpendicular direction by additional cyanide linkages (Figure 10a) [115,116]. When Gd^3+^ ion was used as the lanthanide centre, all the Ru^II^(HAT)-containing networks reveal the strong MLCT photoluminescence in the red range, which was substantially quenched by the insertion of NIR-emitting Nd^3+^ and Yb^3+^ ions. As a result, the strong near-infrared emission was observed, while the residual Ru^II^-based emission was around 100 times weaker than for the GdRu compounds (Figure 10b) [116].

The heteroligand [Os^II^(phen)(CN)_4_]^2−^ ion, built of 5d Os^II^ metal centre, reveals the red MLCT emission, shifted towards lower energy as compared to [Ru^II^(phen)(CN)_4_]^2−^, which was presented for the layered [Gd^III^(H_2_O)_4_(MeOH)][Os^II^(phen)(CN)_4_]_1.5_·4H_2_O framework of a 12-membered metal rings topology [117]. This makes the tetracyanidoosmate(II) an even more efficient sensitizer for NIR-emitting lanthanide(3+) ions than the related tetracyanidoruthenate(II) due to the expected better spectroscopic overlap of Os^2+^-based emission with the absorption peaks of the 4f metal ions. This prediction was checked for the family of cyanido-bridged coordination systems, including hexanuclear Na_2_[Ln^III^(phen)_2_(H_2_O)]_2_[Os^II^(phen)(CN)_4_]_4_·4MeOH·17H_2_O (Ln = Pr, Nd, Er, Yb) clusters, one-dimensional [Ln^III^_2_(bpym)(H_2_O)_7_][Os^II^(phen)(CN)_4_]_3_·MeOH·13H_2_O (Ln = Er, Yb) hybrid chain of squares, and the intricate [Nd^III^_4_(bpym)_2_(H_2_O)_12_(MeOH)][Os^II^(phen)(CN)_4_]_6_·6MeOH·19.5H_2_O coordination layers. For all these species, the NIR lanthanide-based emission was sensitized by the Os^2+^-to-Ln^3+^ energy transfer of distinguishable rates depending on 4f metal ion. The rates of Os^2+^-to-Ln^3+^ energy transfer were found to be an order of magnitude faster than the rates of Ru^2+^-to-Ln^3+^ energy transfer previously observed in similar heterometallic d–f cyanido- bridged networks [117].

The innovative luminescent functionality of heteroligand tetracyanidometallates was shown for the [Ru^II^(^t^Bubpy)(CN)_4_]^2−^ ion bearing the *tert*-butyl derivative of 2,2’-bipyridine [118,119]. This anion embedded in the bimetallic K[Ln^III^(H_2_O)_4_][Ru^II^(^t^Bubpy)(CN)_4_]_2_·8H_2_O (Ln = Pr, Nd, Sm, Eu) chain of {Ln_2_Ru_2_} molecular squares exhibits the orange emission of a typical metal-to-ligand charge transfer (MLCT) origin. The intensity of this photoluminescence detected for the bulk solid sample, and the polymeric film, was reported to be strongly dependent on the presence of gaseous amine molecules, which additionally sensitize the Ru^II^-based emission. Therefore, this material found the analytical application as an efficient and sensitive chemodosimetric detector of biogenic amine odorants including histamine, putrescine, spermidine, and ammonia, indicating a great potential of the polycyanidometallate-based d–f coordination networks in chemical and biochemical sensing based on the spectrofluorometric detection [119].

## 7. Other Cyanide-Containing Building Blocks

**Figure 11:**
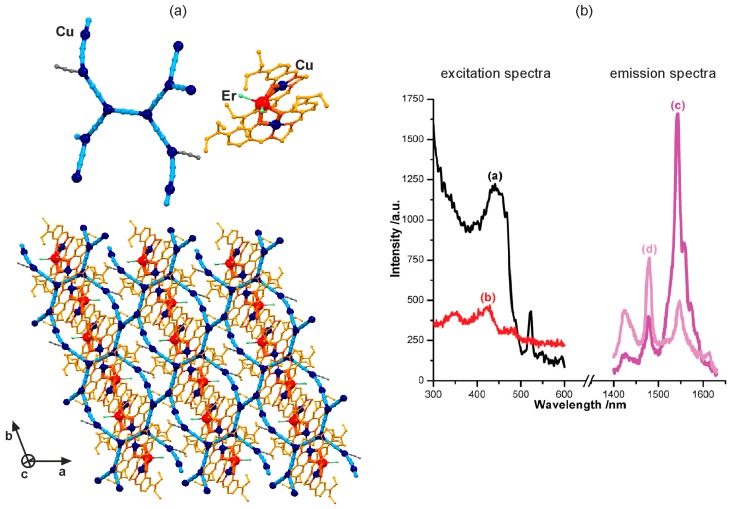
Crystal structure and optical properties of supramolecular [Er^III^Cu^II^_2_(L1)_2_(Cl)_2_][Cu^I^_4_(CN)_5_(MeCN)_4_] (L1 = N,N’-ethylene bis[4-(ethylamino)salicylideneimine anion) network: (**a**) the structural views of two main components, including the fragment of the cyanido-bridged CuI-based layer and the {ErIIICuI2} molecule, and their arrangement in the supramolecular network, (**b**) the room temperature excitation and emission spectra of the reference cyanide-free {ErIII2(H2L)4} molecule (black and purple lines), and the presented supramolecular network (red and light purple lines). Colours for the structural diagram: Er, red; Cu, dark blue; CN−, blue; L1, orange; Cl−, light green. Adapted from Ref. [120] with permission from The Royal Society of Chemistry.

**Figure 12:**
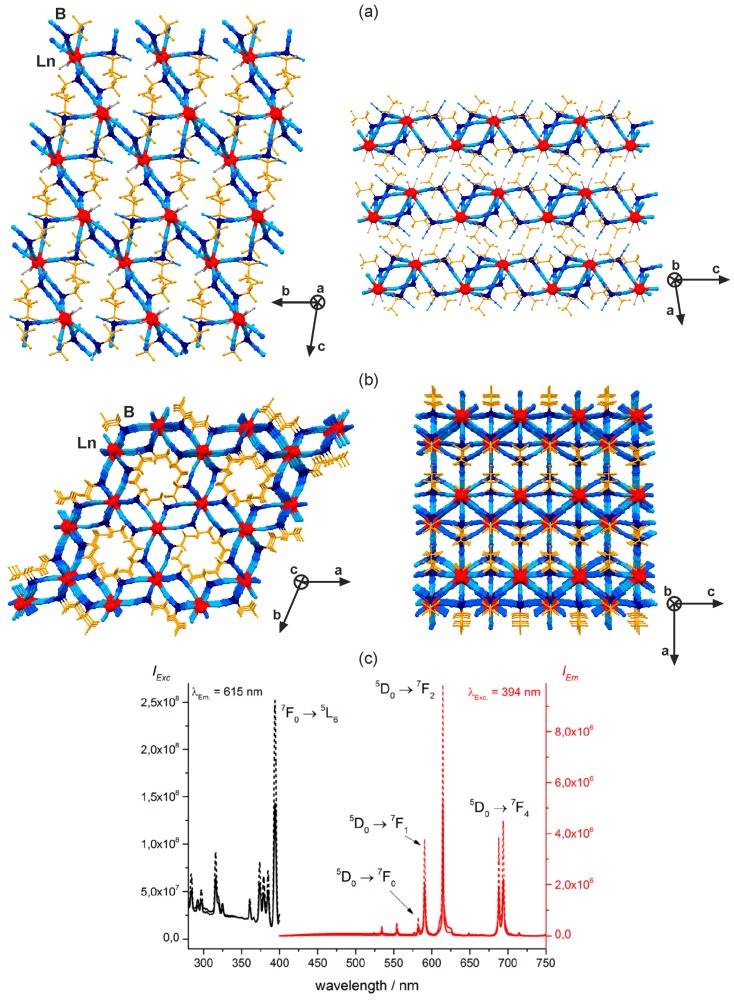
Crystal structure and optical properties of two-dimensional [Ln^III^{C_2_F_5_B(CN)_3_}_3_(H_2_O)_3_](Ln = La, Eu, Ho) coordination polymer (**a**,**c**), and the related anhydrous three-dimensional [Ln^III^{C_2_F_5_B(CN)_3_] (Ln = La, Eu, Ho) coordination network (**b**,**c**): (**a**) the views of the single layer, and the arrangement of layers, (**b**) the views of the 3D cyanido-bridged network along *c* and *b* crystallographic axes, (**c**) the room temperature excitation and emission spectra of the 2D LnB layer (solid lines), and 3D LnB network (dotted lines). Colours for the structural diagram: Ln, red; B, dark blue; CN^−^, blue; C_2_F_5_^−^, orange; H_2_O, grey [123]. Reprinted with permission from *Inorg. Chem.* **2017**, *56*, 2278–2286. Copyright 2017 American Chemical Society.

The photoluminescent cyanido-bridged coordination networks incorporating trivalent lanthanide ions were achieved by a few non-typical cyanide-building blocks (Figure 11 and Figure 12, Table 6) [120,121,122,123]. For instance, the solvothermal reaction of a chemically modified salen-type ligand, N,N’-ethylene bis[4-(ethylamino)salicylideneimine] (L1), with erbium(III) chloride and copper(I) cyanide in a non-aqueous methanol-acetonitrile solution resulted in the isolation of an unprecedented [Er^III^Cu^II^_2_(L1)_2_(Cl)_2_][Cu^I^_4_(CN)_5_(MeCN)_4_] supramolecular network. It is composed of the two-dimensional homometallic Cu^I^-based cyanido-bridged layers of a 10-membered metal rings topology with the trinuclear salen-ligand-bridged {Er^III^Cu^II^_2_L1_2_Cl_2_} clusters occupying the interlayer space (Figure 11a) [120]. This material reveals NIR Er^3+^-centred emission under the visible light excitation, slightly weakened when compared with the reference cyanide-free {Er^III^_2_(H_2_L)_4_} molecule due to the partial Er^3+^-to-Cu^2+^ f–d energy transfer (Figure 11b). This work showed that a cuprous cyanide network serves as a stabilizing agent for the formation of unique NIR-emitting {Er^III^Cu^II^_2_L1_2_Cl_2_} clusters, non-isolable under cyanide-free synthetic conditions [120].

The rare organometallic heteroligand dicyanidoiridate(III) anion, [Ir^III^(ppy)_2_(CN)_2_]^−^, with four coordination sites blocked by two anions of 2-phenylpyridine (ppy), was also tested as a novel cyanide-containing metal complex for d–f photoluminescent materials [121]. The self-assembly of this specific dicyanidometallate with the excess of lanthanide(3+) ions resulted in a series of cyanido-bridged tetranuclear [Ln^III^(H_2_O)_2_(MeCN)(NO_3_)_2_]_2_[Ir^III^(ppy)_2_(CN)_2_]_2_·4MeCN (Ln = La, Pr, Nd), {N(PPh_3_)_2_}_2_[Eu^III^_2_(H_2_O)(NO_3_)_6_][Ir^III^(ppy)_2_(CN)_2_]_2_·5MeCN, and [Gd^III^(NO_3_)_2_(H_2_O)_2_]_2_[Ir^III^(ppy)_2_ (CN)_2_]_2_ molecules which structural details depend on the attached lanthanide(3+) ions. In all these materials, the broad green [Ir^III^(ppy)_2_(CN)_2_]^−^-based emission was detected, the strongest for the UV-emissive Gd^3+^, and much weaker for visible-light-emissive Eu^3+^, and NIR-emitting Nd^3+^. It suggested the presence of Ir^3+^-to-Eu^3+^ and Ir^3+^-to-Nd^3+^ energy transfer processes, but the sensitized Eu^3+^ or Nd^3+^ could not be detected indicating the additional quenching effect, presumably involving the water molecules existing in the crystal structure [121].

Non-heterometallic, however, cyanido-bridged photoluminescent coordination networks were obtained by combining trivalent lanthanide ions with inorganic tetracyanidoborate, [B^III^(CN)_4_]^−^ anionic building block, and its heteroligand pentafluoroethyltricyanidoborate(III), {[C_2_F_5_B^III^(CN)_3_]^−^ derivative [122,123]. The rare-earth metal oxide or hydroxides mixed with tetracyanidoboronic acid, H[B(CN)_4_]·*n*H_2_O produced the cyanido-bridged dinuclear [Ln^III^(H_2_O)_7_][B(CN)_4_]·2{B(CN)_4_}(Ln = Tb, Dy) molecules where tetracyanidoborate(III) serves as a ligand, and a crystallization counterion. The TbB and DyB compounds reveal the intense sharp peaks of their characteristic visible photoluminescence, realized by direct intrashell f–f excitation, and interconfigurational f–d excitation pathways [121]. Thus, [B^III^(CN)_4_]^−^ serves only as a supporting inorganic ligand without the significant emission or sensitizing effect. Much more pronounced role in the photoluminescent effect is played by its structurally modified derivative, that is pentafluoroethyltricyanidoborate(III) ion, [C_2_F_5_B^III^(CN)_3_]^−^ [123]. This anionic building block combined with lanthanide(3+) ions resulted in the formation of a hydrated cyanido-bridged [Ln^III^{C_2_F_5_B(CN)_3_}_3_(H_2_O)_3_](Ln = La, Eu, Ho) layered framework showing the topology of cross-linked {Ln_2_B_2_}-square-based chains (Figure 12a). Upon thermal dehydration, this material could be transformed into the anhydrous three-dimensional cyanido-bridged [Ln^III^{C_2_F_5_B(CN)_3_}](Ln = La, Eu, Ho) hexagonal network built of nine-coordinated Ln^3+^ ions which coordination sphere is fully occupied by cyanide ligands of bridging [C_2_F_5_B^III^(CN)_3_]^−^ anions (Figure 12b). The LaB anhydrous network exhibits the broad greenish-blue UV-light-induced photoluminescence of tricyanidoborate(III) anions. This emission is also observed for HoB analogues but the intense reabsorption effect was detected as the several characteristic f–f lines situated within the broad emission band of the anion. It suggested the lack of efficient [C_2_F_5_B^III^(CN)_3_]^−^- to-Ho^3+^ energy transfer. On the contrary, the sensitization effect was found for the hydrated and anhydrous EuB networks that show the strong 4f-metal-centred emission, and the complete quenching of the borate-based luminescence (Figure 12c). Due to the removal of water molecules, which presence typically decreases the lanthanide(3+) emission, the anhydrous phase reveals almost three times stronger photoluminescence than the related hydrated species. All these results proved that pentafluoroethyltricyanidoborate(III) anion is a promising luminescent cyanide building block for sensitization of the visible photoluminescence of lanthanide(3+) ions.

## 8. Conclusions

We have presented a detailed overview of lanthanide photoluminescence in coordination networks based on polycyanidometallates. To best of our knowledge, this is the first systematic catalogue of the state of the art in the investigation of photoluminescent d–f cyanido-bridged systems.

Selected groups of polycyanidometallates are suitable for construction of photoluminescent coordination frameworks that can explore intrinsic optical properties of lanthanide(3+) ions. Among them, the dicyanidometallates of Au^I^ and Ag^I^ are luminescent in the visible range due to the metal-to-ligand charge transfer (MLCT) transitions. Such emission, found in 4d/5d–4f coordination polymers, and facilitated by the presence of Au–Au interactions, was shown to be dependent on pressure and temperature. Dicyanidometallates are good sensitizers for visible emission of 4f-metal ions, and the resulting emission can be tuned by external stimuli such as pressure, temperature, or light excitation. Thus, the multi-coloured tunable photoluminescence is the main application of [M^I^(CN)_2_]-based d–f systems, taking also into account the possible insertion of organic ligands, introducing additional energy transfer pathways, or separate emission components. Similar luminescence was achievable for tetracyanidoplatinate(II) ions revealing the visible emission due to the metal-to-metal-to-ligand charge transfer (MMLCT) transitions related to the Pt–Pt interactions. This emission is dependent on temperature, pressure, and light polarization due to the anisotropic character of these square planar polycyanido- metallates. [Pt^II^(CN)_4_]^2−^ is a good sensitizer for some lanthanide(3+) ions, including Eu^3+^, Sm^3+^, or Tb^3+^. The tetracyanidoplatinate(II)-based systems offer a structural diversity due to the ancillary organic ligands, modifying the 4f-metal-centred emission.

The octahedral hexacyanidometallates of Co^III^ and Cr^III^ are luminescent in the red and NIR ranges, respectively. They can enhance NIR lanthanide(3+) emission by the energy transfer. [Co^III^(CN)_6_]^3−^ can also sensitize visible 4f-metal-centred luminescence. They offer six cyanides to create extended networks, and leave the coordination sites on lanthanides that can be occupied by organic ligands. Thus, various optical functionalities were achieved, as exemplified by white light emission of Dy^III^–Co^III^ compound, excitation- and composition-switchable multi-coloured emission in Eu^III^–Tb^III^–Co^III^ chains, and dual-donor NIR emission in Nd^III^–Cr^III^ and Yb^III^–Co^III^ networks.

Despite their non-luminescent character, octacyanidometallates of Mo^IV/V^ and W^IV/V^ were fruitfully applied in the synthesis of photoluminescent d–f coordination systems. Due to the optical transparency in the Vis–NIR range, their cyanido-bridged networks with lanthanides could reveal the rich spectrum of intrinsic properties of 4f metal ions, and the sensitization effects on supporting organic ligands. [M(CN)_8_]*^n^*^−^ ions offer a great structural flexibility, so a number of d–f coordination networks could be rationally designed. As a result, such attractive functionalities as thermal switching of blue–red emission in Eu^III^–W^V^ helices, excitation-tunable green–red luminescence in Tb^III^–W^V^ layers, or humidity sensor based on red emissive Eu^III^–Mo^IV^ network, were reported.

The heteroligand tetracyanidometallates of Ru^II^ and Os^II^, [M^II^(CN)_4_(L)]^2−^ are photoluminescent in the visible range due to the metal-to-ligand charge transfer (MLCT) transition. These anions are good sensitizers for NIR-emitting lanthanide(3+) ions. Their emission energy can be tuned by the blocking ligand L, thus, the selection of appropriate organic part governs the efficiency of the Ru^2+^/Os^2+^-to-Ln^3+^ energy transfer process. Metal center also plays a vital role, and the Os^II^-based cyanide complexes were proved to be better sensitizers for NIR emission than the analogous Ru^II^ species. The ancillary organic ligands can be, here, inserted to amend the structural features, and facilitate the characteristics of energy transfer. Lately, the heteroligand tetracyanidoruthenate(II) embedded in d–f cyanido-bridged networks has successfully been applied in the chemodosimetric detection of biogenic amines utilizing a selective sensitization of its visible MLCT emission.

Among other cyanide-containing building blocks, the cuprous cyanide networks was found to stabilize NIR-emitting salen-based Er^III^–Cu^III^ clusters, while the green emissive heteroligand dicyanidoiridate(III) bearing two blocking bidentate ligands were tested as possible sensitizers for lanthanide(3+) luminescence. In addition, the inorganic tetracyanidoborate anion was used for the construction of photoluminescent Tb^III^- and Dy^III^-based molecules, however, without a strong impact on the emission properties. On the contrary, its modified analogue, pentafluoroethyltri- cyanidoborate(III) ion, [C_2_F_5_B^III^(CN)_3_]^−^ is greenish-blue emissive, and efficiently sensitizes the Eu^3+^ luminescence within the cyanido-bridged two- or three-dimensional coordination networks.

In summary, we have gathered all reported photoluminescent polycyanidometallate-based coordination systems incorporating lanthanide ions. We have shown that cyanide metal complexes are useful for the preparation of emissive solids revealing such functionalities as lanthanide- centred Vis–NIR photoluminescence, sensitized through metal-to-metal and/or ligand-to-metal energy transfer effects, and multi-coloured emission tuned by external stimuli of excitation wavelength, temperature, and pressure. There is still several challenges in this area, including the detailed investigation of structural and electronic features governing the sensitization process involving cyanide complexes, and the preparation of novel luminescent polycyanidometallates of improved optical properties, and affinity to lanthanide(3+) ions. The particularly promising future work comprises of searching for multifunctional photoluminescent materials that can exhibit the undiscovered physical cross-effects, as polycyanidometallate-based heterometallic coordination networks showed a diversity of physical properties, such as magnetic anisotropy leading to slow magnetic relaxation, magnetic ordering, spin transitions, catalytic activity, ferroelectricity, and ionic conductivity, that in some cases lead to the unprecedented physical phenomena [38,39,40,41,42,50].

## Figures and Tables

**Table 1:** Photoluminescent dicyanidometallate-based compounds containing lanthanide ions.

Compound	Structural Type ^1^	Luminescent Property	Ref.
[Ln^III^(H_2_O)_3_][M^I^(CN)_2_]_3_ (Ln = La, Ce, Pr, Nd, Sm, Eu, Gd, Tb, Dy; M = Ag, Au)	3D network	Visible Ln^3+^ (Pr, Eu, Tb, Dy) emission with pressure- and temperature-dependent [M(CN)_2_]^−^ -to-Ln^3+^ ET.^2^ UV-Vis pressure- and temperature- tunable emission of [M(CN)_2_]^−^ (MLCT) ^3^ for LnM (Ln = La, Nd, Gd). UV-Vis mixed Ln^3+^ (Ce, Sm) and [Au(CN)_2_]^−^ emission.	[55,56,57,58,59,60,61,62,63,64,65,66,67,68,69]
(^n^Bu_4_N)_2_[Ln^III^(NO_3_)_4_][Au^I^(CN)_2_] (Ln = Ce, Nd, Sm, Gd, Eu, Tb, Dy)	1D zig-zag chain	UV-violet [Au(CN)_2_]^−^ emission for NdAu and GdAu. Excitation- and composition- tunable visible [Au(CN)_2_]^−^ and Ln^3+^ (Sm, Eu, Tb, Dy) emission in LnAu. UV-violet Ce^3+^ emission in CeAu.	[67,68,69,70]
[Ln^III^(terpy)(H_2_O)(NO_3_)_2_][Au^I^(CN)_2_] ^4^ (Ln = Sm, Eu, Gd, Tb, Dy, Ho, Er, Yb)	0D dinuclear molecule	Visible Ln^3+^ (Sm, Eu, Tb, Dy) and green [Au^I^(CN)_2_]^−^ emission with terpy-to-Ln^3+^ and partial [Au(CN)_2_]^−^ -to-Ln^3+^ ET. Mixed green [Au(CN)_2_]^−^ and red terpy-based emission in GdAu.	[71,72]
[Ln^III^(bbp)_2_][Au^I^(CN)_2_]_3_·2MeCN ^5^ (Ln = Eu, Gd)	0D tetranuclear molecule	Red Eu^3+^ (EuAu) emission with bbp-to-Ln^3+^ ET. Mixed violet [Au(CN)_2_]^−^ and green bbp emission in GdAu.	[73]
[Ln^III^(bbp)(NO_3_)_2_][Au^I^(CN)_2_]·MeCN (Ln = Eu, Gd, Tb)	1D linear chain	Green Tb^3+^ (GdAu) or red Eu^3+^ (EuAu) emission with bbp-to-Ln^3+^ ET. Green bbp-based emission in GdAu.	[73]

^1^ cyanido-bridged skeleton, excluding the Ag–Ag or Au–Au short contacts; ^2^ ET = energy transfer; ^3^ MLCT = metal-to-ligand charge transfer; ^4^ terpy = 2,2’:6’2”-terpyridine; ^5^ bbp = bis(benzimidazole)pyridine.

**Table 2:** Photoluminescent tetracyanidometallate-based compounds containing lanthanide ions.

Compound	Structural Type ^1^	Luminescent Property	Ref.
[Ln^III^(H_2_O)_6_]_2_[Pt^II^(CN)_4_]·2{Pt^II^(CN)_4_}·9H_2_O (Ln = La–Lu)	2D 6-membered metal rings	Visible polarization-dependent emission from 1D [Pt(CN)_4_]^2−^ stacks (MMLCT) ^2^.	[74,75,76]
[Ln^III^_2_(H_2_O)_11_][Pt^II^(CN)_4_]·2{Pt^II^(CN)_4_}·10H_2_O (Ln = Sm, Eu)	2D 6-membered metal rings	Polarization-dependent green to orange emission from 1D [Pt(CN)_4_]^2−^ stacks. Red Eu^3+^ (EuPt) or Sm^3+^ (SmPt) emission with temperature- and pressure-dependent [Pt(CN)_4_]^2−^-to-Ln^3+^ ET.^3^	[74,77,78,79]
[Er^III^(H_2_O)_5_(SO_4_)]_2_[Pt^II^(CN)_4_]·{Pt^II^(CN)_4_}·1.5H_2_O	2D 6-membered metal rings	Polarization-dependent green to orange emission from 1D [Pt(CN)_4_]^2−^ stacks.	[74]
[Ln^III^(tpp)(dmf)_n_]_2_[M^II^(CN)_4_] ^4^ (Ln = Er, Yb; M = Ni, Pd, Pt)	0D trinuclear molecule	Red tpp, and NIR Er^3+^ (ErM) or Yb^3+^ (YbM) emission.	[80]
[Eu^III^(terpy)(H_2_O)_2_(NO_3_)][Pt^II^(CN)_4_]·MeCN ^5^	1D zig-zag chain	Red Eu^3+^ emission with terpy-to-Eu^3+^ and [Pt(CN)_4_]^2−^-to-Eu^3+^ ET.	[81,82]
[Eu^III^(terpy)(H_2_O)_3_]_2_[Pt^II^(CN)_4_]_3_·2H_2_O	1D ladder chain	Red Eu^3+^ emission with terpy-to-Eu^3+^ and [Pt(CN)_4_]^2−^-to-Eu^3+^ ET.	[82]
[Eu^III^(terpy)(H_2_O)_2_(CH_3_COO)_2_]_2_·{Pt^II^(CN)_4_}·3H_2_O	0D ionic salt	Red Eu^3+^ emission with terpy-to-Eu^3+^ ET; additional emission from [Pt(CN)_4_]^2−^.	[82]
[Eu^III^(dmf)_2_(terpy)(H_2_O)_2_(NO_3_)]·{Pt^II^(CN)_4_} ^6^	0D ionic salt	Red Eu^3+^ emission with terpy-to-Eu^3+^ ET; additional emission from [Pt(CN)_4_]^2−^.	[83]
[Ln^III^(dmso)_4_(H_2_O)_3_][M^II^(CN)_4_]·0.5{M^II^(CN)_4_}·2H_2_O ^7^ (Ln = Eu, Tb; M = Pd, Pt)	0D ionic salt	Red Eu^3+^ (EuM) or green Tb^3+^ (TbM) emission with direct f-f excitation.	[84]
[Ln^III^(dmso)_2_(phen)(H_2_O)_3_]_2_[Pt^II^(CN)_4_]·2{Pt^II^(CN)_4_}·2(phen)·4H_2_O ^8^ (Ln = Eu, Tb, Yb)	0D trinuclear molecule	Red Eu^3+^ (EuPt) or green Tb^3+^ (TbPt) emission with phen-to-Ln^3+^ ET.	[85]
[Ln^III^(dmf)_3_(phen)(H_2_O)_2_(NO_3_)]·{Pt^II^(CN)_4_} (Ln = La, Eu, Tb)	0D ionic salt	Red Eu^3+^ (EuPt) or green Tb^3+^ (TbPt) emission with phen-to-Ln^3+^ ET.	[85]
[Ln^III^(dmf)_3_(2,2’-bpy)(H_2_O)_2_(NO_3_)]·{Pt^II^(CN)_4_} ^9^ (Ln = La, Sm, Eu, Tb)	0D ionic salt	Red Eu^3+^ (EuPt) or green Tb^3+^ (TbPt) emission with 2,2’-bpy-to-Ln^3+^ ET.	[85]
K_2_[Tb^III^(H_2_O)_4_][Pt^II^(CN)_4_]_2_·{Au^I^(CN)_2_}·2H_2_O	2D 8-membered metal rings	Green Tb^3+^ emission with {Au_2_Pt_4_}-to-Tb^3+^ ET; additional weak blue {Au_2_Pt_4_} emission.	[86]
[Tb^III^(2,2’-bpy)(H_2_O)_4_][Pt^II^(CN)_4_][Au^I^(CN)_2_]·1.5(2,2’-bpy)·2H_2_O	0D trinuclear molecule	Green Tb^3+^ emission with {Au_2_Pt_2_}-to-Tb^3+^ ET; additional weak violet 2,2’-bpy emission.	[86]
[Tb^III^(terpy)(H_2_O)_2_(NO_3_)][Pt^II^(CN)_4_]·*n*(solvent)	1D zig-zag chain	Green Tb^3+^ emission with terpy-to-Tb^3+^ ET; additional blue [Pt(CN)_4_]^2−^ emission.	[87]
[Tb^III^(terpyCl)(H_2_O)_2_(NO_3_)][Pt^II^(CN)_4_]·2.5H_2_O ^10^	1D zig-zag chain	Green Tb^3+^ emission with terpyCl-to-Tb^3+^ ET; additional blue [Pt(CN)_4_]^2−^ emission.	[87]
[Tb^III^(terpy)(H_2_O)_2_(CH_3_COO)_2_]_2_·{Pt^II^(CN)_4_}·4H_2_O	0D ionic salt	Green Tb^3+^ emission with terpy-to-Tb^3+^ and CH_3_COO^−^-to-Tb^3+^ ET.	[87]
[Tb^III^_2_(terpy)_2_(H_2_O)_2_(CH_3_COO)_5_]·{Pt^II^(CN)_4_}·7H_2_O	0D ionic salt	Green Tb^3+^ emission with terpy-to-Tb^3+^ and CH_3_COO^−^-to-Tb^3+^ ET.	[87]
K[Ln^III^(H_2_O)_6_]_2_[Pt^II^(CN)_4_]_3_·{Pt^II^(CN)_4_}·5.5H_2_O (Ln = La, Pr, Nd)	2D honeycomb	Green emission from 1D [Pt(CN)_4_]^2−^ stacks (MMLCT).	[88]

^1^ cyanido-bridged skeleton, excluding the Pt–Pt and the analogus M–M short contacts; ^2^ MMLCT = metal-metal-to-ligand charge transfer; ^3^ ET = energy transfer; ^4^ terpy = 2,2’:6’2”-terpyridine; ^5^ tpp = tetraphenylporphyrinate dianion; ^6^ dmf = N,N-dimethylformamide; ^7^ dmso = dimethylsulfoxide; ^8^ phen = 1,10-phenanthroline; ^9^ 2,2’-bpy = 2,2’-bipyridine; ^10^ terpyCl = 4’-chloro-2,2’:6’2”-terpyridine.

**Table 3:** Photoluminescent hexacyanidometallate-based compounds containing lanthanide ions.

Compound	Structural Type	Luminescent Property	Ref.
[Gd^III^(H_2_O)_2_][M^III^(CN)_6_]·2H_2_O (M = Cr, Co)	3D network	Red Co^3+^(GdCo) or NIR Cr^3+^ (GdCr) emission.	[89,90,91]
[Eu^III^(H_2_O)_2_][Co^III^(CN)_6_]·2H_2_O	3D network	Red Eu^3+^ emission with Co^3+^-to-Eu^3+^ ET ^1^.	[92,93]
[Ln^III^(dmf)_4_(H_2_O)_2_][Cr^III^(CN)_6_]·*n*H_2_O (Ln = Nd, Gd, Yb) ^2^	1D chain	NIR Nd^3+^ (NdCr) or Yb^3+^ (YbCr) emission with Cr^3+^-to-Nd^3+^ and Cr^3+^-to-Yb^3+^ ET; NIR Cr^3+^ emission (GdCr).	[94]
[Ln^III^(dmf)_4_(H_2_O)_3_][Co^III^(CN)_6_]·*n*H_2_O (Ln = Nd, Gd, Yb)^2^	0D dinuclear molecule	NIR Nd^3+^ (NdCo) or Yb^3+^ (YbCo) emission with Co^3+^-to-Nd^3+^ and Co^3+^-to-Yb^3+^ ET; red Co^3+^ emission (GdCo).	[94,95]
[Dy^III^(3-OHpy)_2_(H_2_O)_4_][Co^III^(CN)_6_]·H_2_O ^3^	1D zig-zag chain	White light Dy^3+^ emission with direct f-f excitation, Co^3+^-to-Dy^3+^ and 3-OHpy-to-Dy^3+^ ET.	[96]
[Eu^III^_x_Tb^III^_1-x_(3-OHpy)_2_(H_2_O)_4_][Co^III^(CN)_6_]·H_2_O^3^	1D zig-zag chain	Excitation and Eu/Tb ratio switching from red Eu^3+^ to green Tb^3+^ emission with Co^3+^-to-Ln^3+^ ET.	[97]
[Dy^III^(4-OHpy)_2_(H_2_O)_3_][Co^III^(CN)_6_]·0.5H_2_O ^4^	2D 6-membered metal rings	Excitation switching from yellow Dy^3+^ emission with Co^3+^-to-Dy^3+^ ET, to greenish- blue 4-OHpy emission.	[98]
[Nd^III^(pmmo)_2_(H_2_O)_3_][Cr^III^(CN)_6_] ^5^	2D square grid	NIR Nd^3+^ emission with Cr^3+^-to-Nd^3+^ and pmmo-to-Nd^3+^ ET.	[99]
[Yb^III^(3-pyone)_2_(H_2_O)_2_][Co^III^(CN)_6_] ^6^	1D chain	NIR Yb^3+^ emission with Co^3+^-to-Yb^3+^ and 3-pyone-to-Yb^3+^ ET.	[100]

^1^ ET = energy transfer; ^2^ dmf = N,N-dimethylformamide; ^3^ 3-OHpy = 3-hydroxypyridine; ^4^ 4-OHpy = 4-hydroxypyridine; ^5^ pmmo = pyrimidine N-oxide; ^6^ 3-pyone = 3-pyridone.

**Table 4:** Photoluminescent octacyanidometallate-based compounds containing lanthanide ions.

Compound	Structural type	Luminescent property	Ref.
[Eu^III^(H_2_O)_5_][M^V^(CN)_8_] (M = Mo, W)	2D square grid	Red Eu^3+^ emission with O-Eu (EuMo) or O-W (EuW) LMCT^1^ and direct f-f excitation.	[101]
[Tb^III^(H_2_O)_5_][M^V^(CN)_8_] (M = Mo, W)	2D square grid	Green Tb^3+^ emission with Tb-based f-d and f-f excitation.	[101,102]
[Eu^III^_0.5_Gd^III^_0.5_(H_2_O)_5_][W^V^(CN)_8_]	2D square grid	Red Eu^3+^ emission with dominant LMCT (O-Eu or W-related) excitation.	[103]
[Eu^III^_0.5_Tb^III^_0.5_(H_2_O)_5_][W^V^(CN)_8_]	2D square grid	Red Eu^3+^ and green Tb^3+^ emission with LMCT (O-Eu or W-related) excitation and additional Tb^3+^-to-Eu^3+^ ET.^2^	[103]
[Sm^III^_0.5_Tb^III^_0.5_(H_2_O)_5_][W^V^(CN)_8_]	2D square grid	Green Tb^3+^ emission with Tb-based f-d and f-f excitation.	[103]
[Nd^III^(phen)_2_(dmf)_2_(H_2_O)][Mo^V^(CN)_8_]·2H_2_O^3,4^	1D chain	NIR Nd^3+^ emission with phen-to-Nd^3+^ ET.	[104]
[Nd^III^(phen)(dmf)_5_][M^V^(CN)_8_]·*x*H_2_O (M = Mo, W)	1D chain	NIR Nd^3+^ emission with direct f-f excitation, phen-to-Nd^3+^ ET.	[104]
[Ln^III^_2_(mpca)_2_(MeOH)_2_(H_2_O)_6_][Mo^IV^(CN)_8_]·*x*MeOH^5^ (Ln = Nd, Eu, Tb)	3D hybrid I^2^O^1^ network	Red Eu^3+^ (EuMo), green Tb^3+^ (TbMo) and NIR Nd^3+^ emission with mpca-to-Ln^3+^ ET, and humidity-dependent intensity of Eu^3+^ emission.	[105,106]
[Eu^III^(*i*Pr-pybox)(dmf)_4_][W^V^(CN)_8_]·dmf·8H_2_O^6^	1D helical chain	Thermal switching between red Eu^3+^ and blue *i*Pr-pybox emission.	[107]
[Ln^III^(*i*Pr-pybox)(dmf)_4_][W^V^(CN)_8_]·dmf·4H_2_O (Ln = Nd, Gd)	1D helical chain	NIR Nd^3+^ emission with pybox-to-Nd^3+^ ET(NdW); red *i*Pr-pybox emission (GdW).	[108]
[Ln^III^(ind-pybox)(dmf)_4_][W^V^(CN)_8_]·5MeCN·4MeOH^7^ (Ln = Nd, Gd)	1D helical chain	NIR Nd^3+^ emission with direct f-f excitation and pybox-to-Nd^3+^ ET (NdW); red ind-pybox emission (GdW).	[109]
[Tb^III^(box)_2_(dmf)_2_][W^V^(CN)_8_]·H_2_O^8^	2D mixed 4- and 8-membered metal rings	Excitation switching between green Tb^3+^ and red box emission.	[109]
[Ln^III^(box)_n_(dmf)_m_][Mo^V^(CN)_8_]·*x*(solvent) (Ln = Ce–Dy, n = 2, m = 2; Ln = Ho–Yb, n = 1, m = 3)	2D mixed 4- and 8-membered metal rings	Visible Ln^3+^(Pr, Sm, Eu, Ho) or NIR Ln^3+^ (Pr, Nd, Sm, Ho, Yb) emission with direct f-f excitation and box-to-Ln^3+^ ET.	[110]

^1^ LMCT = ligand-to-metal charge transfer; ^2^ ET = energy transfer; ^3^ phen = 1,10-phenanthroline; ^4^ dmf = N,N-dimethylformamide; ^5^ mpca = 5-methyl-2-pyrazine carboxylic acid; ^6^ *i*Pr-pybox = (*SS*)- or (*RR*)-2,2’-(2,6-pyridinediyl)bis(4-isopropyl-2-oxazoline); ^7^ ind-pybox = (*SRSR*)- or (*RSRS*)-2,6-bis[8H-indeno[1,2-d]oxazolin-2-yl]pyridine; ^8^ box = 2,2’-bis(2-oxazoline).

**Table 5:** Photoluminescent coordination compounds containing lanthanide ions and heteroligand tetracyanidometallates.

Compound	Structural type	Luminescent property	Ref.
K[Ln^III^(H_2_O)_n_][Ru^II^(2,2’-bpy)(CN)_4_]_2_·9H_2_O^1^ (*n* = 7, Ln = Pr; *n* = 6, Ln = Er, Yb)	0D trinuclear molecule	NIR Ln^3+^ (Ln = Pr, Er, Yb) emission with Ru^2+^-to-Ln^3+^ ET.^2^	[111,112]
[Ln^III^(H_2_O)_4_]_2_[Ru^II^(2,2’-bpy)(CN)_4_]_3_·*n*H_2_O (Ln = Gd, Nd)	2D mixed 4- and 12-membered metal rings	NIR Nd^3+^ emission (NdRu) with Ru^2+^-to-Nd^3+^ ET. Yellow [Ru(2,2’-bpy)(CN)_4_]^2−^ emission in GdRu (MLCT).^3^	[112]
[Ln^III^(H_2_O)_5_(NO_3_)][Ru^II^(bpym)(CN)_4_]^5^ (Ln = Nd, Sm, Gd)	1D helical chain	NIR Nd^3+^ emission (NdRu) with Ru^2+^-to-Nd^3+^ ET. Red [Ru(bpym)(CN)_4_]^2−^ emission in GdRu (MLCT).	[113]
[Ln^III^_2_(H_2_O)_7.5_(NO_3_)_1.5_][Ru^II^(bpym)(CN)_4_]_2_·5.5H_2_O (Ln = Er, Yb)	2D cross-linked square-based chains	NIR Ln^3+^ (Ln = Er, Yb) emission with Ru^2+^-to-Ln^3+^ ET.	[113]
[Ln^III^(H_2_O)_5_(NO_3_)]_2_[{Ru^II^(CN)_4_}_2_ (bpym)]·3H_2_O (Ln = Nd, Sm)	1D hybrid ladder chain	NIR Nd^3+^ emission in NdRu with Ru^2+^-to-Nd^3+^ ET.	[113]
[Ln^III^_1.5_(H_2_O)_7_][{Ru^II^(CN)_4_}_2_(bpym)]·(NO_3_)·*n*H_2_O (Ln = Eu, Gd, Yb)	3D hybrid I^2^O^1^ network	NIR Yb^3+^ emission (YbRu) with Ru^2+^-to-Yb^3+^ ET. Red [{Ru^II^(CN)_4_}_2_(bpym)]^4−^ emission in GdRu (MLCT).	[113]
K_2_[Ln^III^(phen)_2_(H_2_O)]_2_[Ru^II^(phen)(CN)_4_]_4_·*n*(solvent)^6^ (Ln = Pr, Nd, Er, Yb)	0D hexanuclear	NIR Ln^3+^ (Ln = Nd, Er, Yb) emission with Ru^2+^-to-Ln^3+^ ET.	[114]
[Ln^III^(phen)(H_2_O)_3_]_2_[Ru^II^(phen)(CN)_4_]·14H_2_O (Ln = Nd, Gd, Er, Yb)	2D honeycomb	NIR Ln^3+^ (Ln = Nd, Er, Yb) emission with Ru^2+^-to-Ln^3+^ ET. Red [Ru(phen)(CN)_4_]^2−^ emission in GdRu (MLCT).	[114]
[Ln^III^(terpy)(H_2_O)_3_]_2_[Ru^II^(phen)(CN)_4_]_3_·*n*H_2_O^7^ (Ln = Pr, Nd, Er, Yb)	1D ladder chain	NIR Ln^3+^ (Ln = Nd, Er, Yb) emission with Ru^2+^-to-Ln^3+^ ET.	[114]
[Ln^III^_2_(bpym)(H_2_O)_7_][Ru^II^(phen)(CN)_4_]_3_·*n*solvent (Ln = Nd, Er, Yb)	1D hybrid chain of squares	NIR Ln^3+^ (Ln = Nd, Er, Yb) emission with Ru^2+^-to-Ln^3+^ ET.	[114]
[Nd^III^(H_2_O)_5_]_2_[{Ru^II^(CN)_4_}_3_(HAT)]·13H_2_O^4^	3D pillared network	NIR Nd^3+^ emission with Ru^2+^-to-Nd^3+^ ET.	[115]
[Ln^III^(H_2_O)_5_]_2_[Ru^II^(HAT)(CN)_4_]·*n*H_2_O (Ln = Nd, Sm, Eu, Gd)	2D hybrid of 12-membered metal rings	NIR Nd^3+^ emission (NdRu) with Ru^2+^-to-Nd^3+^ ET. Red [Ru(bpym)(CN)_4_]^2−^ emission in GdRu (MLCT).	[116]
[Yb^III^_2_(H_2_O)_9_(NO_3_)_2_][Ru^II^(HAT)(CN)_4_]·2(NO_3_)·6.5H_2_O	1D ladder chain	NIR Yb^3+^ emission with Ru^2+^-to-Yb^3+^ ET.	[116]
[Yb^III^_3_(H_2_O)_2_(NO_3_)][{Ru^II^(CN)_4_}_2_(HAT)]_2_·20H_2_O	2D hybrid 6-membered metal rings	NIR Yb^3+^ emission with Ru^2+^-to-Yb^3+^ ET.	[116]
[Ln^III^(H_2_O)_4_]_2_[{Ru^II^(CN)_4_}_3_(HAT)]·13H_2_O^4^ (Ln = Nd, Gd, Yb)	3D pillared network	NIR Ln^3+^ (Ln = Nd, Yb) emission with Ru^2+^-to-Ln^3+^ ET.	[116]
[Gd^III^(H_2_O)_4_(MeOH)][Os^II^(phen)(CN)_4_]_1.5_·4H_2_O	2D 12-membered metal rings	Red [Os(phen)(CN)_4_]^2−^ emission (MLCT).	[117]
Na_2_[Ln^III^(phen)_2_(H_2_O)]_2_[Os^II^(phen)(CN)_4_]_4_·4MeOH·17H_2_O (Ln = Pr, Nd, Er, Yb)	0D hexanuclear	NIR Ln^3+^ (Ln = Nd, Er, Yb) emission with Os^2+^-to-Ln^3+^ ET.	[117]
[Ln^III^_2_(bpym)(H_2_O)_7_][Os^II^(phen)(CN)_4_]_3_·MeOH·13H_2_O (Ln = Er, Yb)	1D hybrid chain of squares	NIR Ln^3+^ (Ln = Er, Yb) emission with Os^2+^-to-Ln^3+^ ET.	[117]
[Nd^III^_4_(bpym)_2_(H_2_O)_12_(MeOH)] [Os^II^(phen)(CN)_4_]_6_·6MeOH·19.5H_2_O	2D mixed 3-, 4-, 6-, and 14-membered metal rings	NIR Nd^3+^ emission with Os^2+^-to-Nd^3+^ ET. Red	[117]
K[Ln^III^(H_2_O)_4_][Ru^II^(^t^Bubpy)(CN)_4_]_2_·8H_2_O^8^ (Ln = Pr, Nd, Sm, Eu)	1D chain of squares	Orange [Ru(^t^Bubpy)(CN)_4_]^2−^ emission (MLCT); selective sensitization by gaseous amine molecules.	[118,119]

^1^ 2,2’-bpy = 2,2’-bipyridine; ^2^ ET = energy transfer; ^3^ MLCT = metal-to-ligand charge transfer; ^4^ HAT = hexaazatriphenylene; ^5^ bpym = 2,2’-bipyrimidine; ^6^ phen = 1,10 = phenanthroline; ^7^ terpy = 2,2’:6’2”-terpyridine; ^8 t^Bubpy = 4,4’-di-*tert*-butyl-2,2’-bipyridine.

**Table 6:** Photoluminescent coordination compounds composed of lanthanide ions and other cyanide-containing building blocks.

Compound	Structural type	Luminescent property	Ref.
[Er^III^Cu^II^_2_(L1)_2_(Cl)_2_][Cu^I^_4_(CN)_5_(MeCN)_4_]^1^	2D Cu-cyanide 10-membered metal rings, and trinuclear {ErCu_2_}^+^ counterions	NIR Er^3+^ emission under the visible excitation.	[120]
[Ln^III^(H_2_O)_2_(MeCN)(NO_3_)_2_]_2_[Ir^III^(ppy)_2_(CN)_2_]_2_·4MeCN^2^ (Ln = La, Pr, Nd)	0D tetranuclear molecule	Green [Ir^III^(ppy)_2_(CN)_2_]^−^ emission in NdIr, weakened by partial Ir^3+^-to-Nd^3+^ ET.^3^	[121]
{N(PPh_3_)_2_}_2_[Eu^III^_2_(H_2_O)(NO_3_)_6_][Ir^III^(ppy)_2_(CN)_2_]_2_·5MeCN	0D tetranuclear molecule	Green [Ir^III^(ppy)_2_(CN)_2_]^−^ emission, weakened by partial Ir^3+^-to-Eu^3+^ ET.^3^	[121]
[Gd^III^(NO_3_)_2_(H_2_O)_2_]_2_[Ir^III^(ppy)_2_(CN)_2_]_2_	0D tetranuclear molecule	Green [Ir^III^(ppy)_2_(CN)_2_]^−^ emission.	[121]
[Ln^III^(H_2_O)_7_][B(CN)_4_]·2{B(CN)_4_} (Ln = Tb, Dy)	0D dinuclear molecule	Green Tb^3+^ (TbB) or yellow Dy^3+^ (DyB) emission under direct f-f, and f-d excitations.	[122]
[Ln^III^{C_2_F_5_B(CN)_3_}_3_(H_2_O)_3_] (Ln = La, Eu, Ho)	2D cross-linked square-based chains	Red Eu^3+^ emission in EuB with [C_2_F_5_B(CN)_3_]^−^-to-Eu^3+^ energy transfer.	[123]
[Ln^III^{C_2_F_5_B(CN)_3_}] (Ln = La, Eu, Ho)	3D network	Greenish-blue [C_2_F_5_B(CN)_3_]^−^ emission in LaB and HoB with reabsorption effect in HoB. Red Eu^3+^ emission in EuB EuB with [C_2_F_5_B(CN)_3_]^−^-to-Eu^3+^ energy transfer.	[123]

^1^ L1 = N,N’-ethylene bis[4-(ethylamino)salicylideneimine] anion; ^2^ ppy = anion of 2-phenylpyridine; ^3^ ET = energy transfer.
